# A tissue-engineered human psoriatic skin model: targeting inflammation and glucose metabolism dysregulation in psoriasis using microneedle patches

**DOI:** 10.3389/fmolb.2026.1830240

**Published:** 2026-06-09

**Authors:** Yasmine Ruel, Fatma Moawad, Sergio Cortez Ghio, Davide Brambilla, Roxane Pouliot

**Affiliations:** 1 Faculté de Pharmacie, Université Laval, Québec City, QC, Canada; 2 Centre de Recherche en Organogénèse Expérimentale de l’Université Laval/LOEX, Axe Médecine Régénératrice, Centre de Recherche du CHU de Québec-Université Laval, Québec City, QC, Canada; 3 Faculté de Pharmacie, Université de Montréal, Montreal, QC, Canada; 4 In Silico Data Science, Québec City, QC, Canada; 5 School of Pharmaceutical Sciences, University of Geneva, Geneva, Switzerland

**Keywords:** inflammation, insulin resistance, microneedles, phloretin, psoriasis

## Abstract

Psoriasis, an inflammatory skin disease, affects nearly 43 million individuals globally, and patients are 59% more likely to have type 2 diabetes. In this study, tissue-engineered psoriatic human skin substitutes were produced using keratinocytes and fibroblasts derived from patients with plaque psoriasis, and were enriched with human T lymphocytes to amplify the inflammation. Tissue-engineered healthy human skin substitutes served as a control. The psoriatic model exhibited type 2 diabetes-like features, including diminished uptake of insulin and glucose, with glucose uptake being approximately eight times lower than in the healthy skin model. Insulin-like growth factor signalling was impaired with lower insulin-like growth factor 1 (IGF-1), and insulin-like growth factor binding protein 2 (IGFBP-2) levels, as well as elevated insulin-like growth factor binding protein 4 (IGFBP-4) levels in the cell culture supernatants. This is the first human skin model to concurrently exhibit both psoriatic inflammation and insulin resistance. Moreover, to assess therapeutic potential, phloretin-loaded microneedle patches were applied for 1 week. They reduced the levels of cytokines involved in both psoriasis and insulin resistance: granulocyte colony-stimulating factor (G-CSF), granulocyte-macrophage colony-stimulating factor (GM-CSF), macrophage migration inhibitory factor (MIF), and interleukin-17A (IL-17A). This anti-inflammatory activity was more pronounced than that of systemic-like methotrexate and methotrexate-loaded microneedle patches. The microneedles loaded with phloretin tended to enhance insulin uptake and reduce IGFBP-4 levels in the supernatants; however, these changes were not statistically significant. Prolonging the treatment period beyond 1 week or combining it with another compound loaded into the microneedles that improves insulin sensitivity may increase the efficacy of this approach by simultaneously addressing psoriasis and type 2 diabetes.

## 1 Introduction

Psoriasis is a chronic, immune-mediated skin disorder characterized by keratinocyte hyperproliferation, epidermal thickening, and inflamed lesions infiltrated by abundant immune cells, including T lymphocytes and dendritic cells (Ogawa et al., 2018; Perera et al., 2012). The Global Psoriasis Atlas estimates that 43 million people are diagnosed with psoriasis on a global scale (Global Psoriasis Atlas, 2026). There are different forms of skin psoriasis such as plaque, guttate, inverse, erythrodermic, pustulous and sebopsoriasis (Crow, 2012; Goldsmith et al., 2012). Plaque psoriasis, also known by its medical term, psoriasis vulgaris, is the most common form, affecting 80%–90% of the patients (Crow, 2012), and is characterized by well-defined inflamed red lesions covered with whitish surface scales. This type of psoriasis is easily recognized by the chronicity of the plaques (Goldsmith et al., 2012). Moreover, many patients develop or already have other diseases in combination with this skin disease. Comorbidities refer to the coexistence of multiple health conditions within the same individual, particularly among those diagnosed with a specific disease such as psoriasis (Oliveira Mde et al., 2015). Genetic studies have identified several loci associated with psoriasis that are also implicated in other autoimmune disorders, including ulcerative colitis, an inflammatory condition affecting the colon and rectum, as well as Crohn’s disease and other forms of inflammatory bowel disease (Zhang et al., 2025). These disorders are considered to be comorbid with psoriasis due to shared immunological pathways (Oliveira Mde et al., 2015). Moreover, individuals with psoriasis frequently exhibit additional systemic conditions such as cardiovascular disease, obesity, metabolic syndrome, dyslipidemia, and type 2 diabetes (Gottlieb et al., 2008; Cheng et al., 2012; Holm and Thomsen, 2019).

A meta-analysis demonstrated that patients with psoriasis are 59% more likely to have type 2 diabetes compared with individuals without psoriasis (reflecting increased prevalence) and they also exhibit a 27% higher risk of transitioning to a diabetic state in the future (reflecting increased incidence) (Armstrong et al., 2013). Insulin resistance, a condition in which cells fail to absorb glucose effectively despite the presence of insulin, plays a central role in the development of both type 2 diabetes and psoriasis. In individuals with type 2 diabetes, the body’s reduced sensitivity to insulin often leads to compensatory hyperinsulinemia. Elevated insulin levels may interact with insulin-like growth factor (IGF) receptors, promoting the proliferation of keratinocytes and fibroblasts, which are key contributors to psoriatic skin changes (Abramczyk et al., 2020). Under normal conditions, insulin and IGF facilitate glucose uptake by binding to their respective receptors on target cells (Haywood et al., 2019; Acosta-Martinez and Cabail, 2022). However, when insulin signalling is impaired, often due to cellular insensitivity, glucose uptake is diminished, resulting in elevated blood glucose concentrations. In response, pancreatic β-cells may increase insulin production, further raising circulating insulin levels (Zeitler and Nadeau, 2020; Boucher et al., 2014; Dludla et al., 2023). Interestingly, a clinical study has reported increased insulin expression in the blood of patients with psoriasis compared with healthy controls, and also in psoriatic patients with diabetes compared with diabetic patients or healthy controls (Brazzelli et al., 2021). These findings suggest a link between insulin dysregulation and psoriatic inflammation.

Psoriasis, a multifactorial and immune-driven skin disorder, continues to challenge clinicians and researchers alike. While therapeutic advances have improved symptom control, the absence of a curative solution underscores the need for innovative delivery systems that not only enhance drug efficacy but also align with patient lifestyles and expectations.

One such innovation lies in the realm of microneedle-based transdermal delivery. These miniature skin-piercing structures offer a compelling alternative to conventional topical and systemic routes. By bypassing the stratum corneum, microneedles enable direct access to the dermal and epidermal layers, where psoriatic inflammation resides. Their minimally invasive nature, combined with the potential for prolonged controlled release positions them as a transformative tool in dermatological therapeutics (Ramadon et al., 2022). Microneedles represent more than a delivery tool; they embody a shift toward localized, patient-centric, and modular therapy. Their ability to accommodate diverse pharmacological agents, from small molecules to biologics and nanocarriers, opens new avenues for personalized psoriasis management. As research progresses, the integration of microneedles into clinical practice may redefine how chronic skin diseases are treated, not by suppressing symptoms alone, but by targeting pathology with precision and purpose (Gowda et al., 2023; Moawad et al., 2025).

Preclinical investigations have demonstrated the versatility of microneedles in psoriasis-like animal models, where both monotherapies and combinations of drugs have been explored. Traditional agents already approved for psoriasis treatment such as methotrexate (Du et al., 2019) and tumor necrosis factor alpha (TNF-α) inhibitors (Korkmaz et al., 2015) have been incorporated into microneedle delivery, showing enhanced local bioavailability and reduced systemic exposure and cell hyperproliferation. In parallel, cyclosporin A, a systemic immunosuppressant, has been formulated for microneedle delivery, offering localized action with a minimized systemic burden (Halder et al., 2022; Jeong et al., 2018). Furthermore, microneedles loaded with interleukin-17 (IL-17) antibodies and MXene-based encapsulation have shown promising anti-inflammatory and antiproliferative outcomes, highlighting the adaptability of this technology to diverse therapeutic agents (Wu et al., 2022). More nuanced approaches involve co-loading strategies. For instance, the pairing of Deucravacitinib, a tyrosine kinase 2 (TYK2) inhibitor, with Calcipotriol, a vitamin D analog, yielded synergistic effects, attenuating epidermal hyperplasia, T helper 17 (Th17) cell differentiation, and inflammatory markers, all while maintaining a favourable safety profile (Wang et al., 2024). Similarly, methotrexate combined with epigallocatechin-3-gallate (EGCG), a bioactive polyphenol, demonstrated dual anti-inflammatory and antiproliferative activity (Bi et al., 2023).

The integration of phloretin, another polyphenolic compound, into biodegradable microneedles led to a reduction in cell proliferation, evidenced by decreased antigen Ki67 expression and epidermal thickness, as well as a decline in inflammation, marked by lower CD3^+^ T lymphocytes infiltration, in an imiquimod (IMQ)-induced psoriatic mouse model. One single patch application with phloretin or methotrexate was comparable to two doses of subcutaneous injections of either compound. These phloretin-loaded microneedles have shown a high biosafety profile (Moawad et al., 2024).

In the preceding study, phloretin was selected for its therapeutically relevant antipsoriatic action. In human psoriatic cell cultures comprising T cells and psoriatic keratinocytes, phloretin exhibited antiproliferative effects comparable to those of methotrexate, while its anti-inflammatory activity was even more pronounced. This polyphenol modulated a broad spectrum of pro-inflammatory cytokines, including monocyte chemoattractant protein-1 (MCP- 1/CCL2), macrophage inflammatory protein-1*α* (MIP-1*α*), granulocyte colony-stimulating factor (G-CSF), granulocyte-macrophage colony-stimulating factor (GM-CSF), interleukin*-*1 *α* (IL-1*α*), interleukin*-*1*β* (IL-1*β*), interleukin*-*6 (IL-6), interleukin*-*17A (IL-17A), and TNF-*α*, while reducing the lymphocyte common antigen (CD45) marker. Cell hyperproliferation was controlled, as evidenced by a significant reduction in the expression of the proliferation markers Ki67 and the proliferating cell nuclear antigen (PCNA) (Ruel et al., 2024).

To investigate the impact of microneedle-based therapies using human cells from patients with psoriasis, *in vitro* patient-derived skin models were developed. The antipsoriatic activity of phloretin-loaded microneedle patches was assessed (Ruel et al., 2025) in parallel with the *in vivo* biosafety assessments (Moawad et al., 2024). Although psoriasis-like animal models such as the imiquimod model mimic central pathological characteristics, namely, keratinocyte hyperproliferation and inflammation, psoriasis is inherently a human disease. In murine models, IL-17 is mainly produced by γδ T cells, which are predominant in this animal model (Cai et al., 2011; Yoshiki et al., 2014; Ramírez-Valle et al., 2015; Gray et al., 2011) and rare in human skin, where αβ T cells dominate and drive the pathogenesis (Schön et al., 2021; Matos et al., 2017). These immunological (Mestas and Hughes, 2004) and structural differences (Brohem et al., 2011) highlight the importance of validating therapies using human psoriatic cell models. Using a tissue-engineered human psoriatic skin model, phloretin-loaded microneedle patches selectively reduced the epidermal thickness in hyperproliferative skin while sparing healthy skin, indicating targeted efficacy. Moreover, the microneedles exhibited transdermal diffusion beyond the patch application site and sustained molecular release for up to 2 weeks. The therapeutic effects were comparable with systemic-like methotrexate and microneedle patches loaded with methotrexate (Ruel et al., 2025).

These microneedle therapies are highly promising and warrant closer investigation. Given the inflammatory nature of psoriasis, it is essential to assess their impact on inflammatory processes. Furthermore, since insulin resistance is linked to inflammation, (Polic et al., 2018), it would be worthwhile to investigate whether insulin resistance, associated with type 2 diabetes, a common comorbidity of psoriasis, can be modelled using the psoriatic patient-derived skin substitutes, and whether the microneedles can influence insulin response. In this research, cell culture supernatants from psoriatic and healthy tissue-engineered human skin models were analyzed to assess inflammatory markers related to psoriasis, as well as glucose levels and the regulation of insulin/insulin-like growth factor signalling in both healthy and psoriatic human skin substitutes. The effects of 1-week microneedle patch treatments loaded with either phloretin or methotrexate were also examined in this context and compared with a systemic-like administration of methotrexate.

## 2 Materials and methods

### 2.1 Evaluated treatments

Microneedle patch treatments were compared with the systemic-like administration of methotrexate, a reference therapy for psoriasis. In the context of this *in vitro* experiment, a systemic-like administration refers to the delivery of a therapeutic molecule into the culture medium of the skin substitutes in order to model a systemic mode of administration, as the culture medium interfaces with the dermal layer and thereby mimicking vascular-mediated systemic delivery. Systemic-like methotrexate was administered at an initial dosage recommended by several health associations; the British Association of Dermatologists’ guidelines for the safe and effective prescribing of methotrexate for skin disease (5–15 mg weekly) (Warren et al., 2016), the National Health Service’s guidance on how and when to take methotrexate for patients with psoriasis (5–10 mg weekly) (National Health Service, 2023), and *the Guidelines on the Use of Methotrexate in Psoriasis* (7.5–22.5 mg weekly) (Warren et al., 2016; Carretero et al., 2010). Based on these recommendations, a systemic-like dosage of 8 mg per week was selected, delivered as three doses of 2.66 mg of methotrexate throughout the week. In the culture medium, this dosage represents a concentration of 734 *μ*M of methotrexate. Many studies also reported antipsoriatic efficacy in tissue-engineered human psoriatic skin substitutes (Ruel et al., 2025; Bélanger et al., 2019; Bouchard et al., 2022). With the microneedle patches, the amount of active compound per patch was 86.24 ± 9.29 *μ*g for methotrexate and 69.46 ± 8.33 *μ*g for phloretin. All microneedle patches were prepared and applied to the skin substitutes following the methodology described in our publication. The same effective doses were maintained (Ruel et al., 2025). The microneedles were composed of biodegradable polymers, poly (lactic-*co*glycolic) acid (PLGA) copolymer and polylactic acid (PLA), which both have the status Generally Recognized as Safe (GRAS) excipients (Wang et al., 2021; DeStefano et al., 2020). The upper part of the patch was formulated with a mix of hyaluronic acid and dextran. One microneedle patch was applied to each skin substitute. After 25 min, the upper part of the patch was removed, leaving the microneedles embedded in the skin for a 1-week treatment. For systemic-like administration, methotrexate was obtained as an industrially synthesized injectable formulation (Injectable USP Methotrexate, 25 mg/mL, Galenova, Saint-Hyacinthe, QC, Canada). For the microneedle fabrication, both methotrexate and phloretin were sourced from Toronto Research Chemicals Inc (TRC, North York, ON, Canada).

### 2.2 Skin biopsies and donors

Human skin cells, fibroblasts and keratinocytes, were obtained in accordance with ethical standards and with approval from the Research Ethics Committee of the Centre Hospitalier Universitaire (CHU) de Québec. Skin biopsies were collected by a dermatologist from donors with plaque psoriasis and by a surgeon from healthy donors. All procedures adhered to the principles outlined in the Declaration of Helsinki. Informed consent was obtained from all participants after providing them with comprehensive information about the study.

Psoriatic skin cells were isolated from lesional skin biopsies of three patients with plaque psoriasis: one female aged 36 years old and two males aged 39 and 65 years old (N = 3 donors with psoriasis, PSO1, PSO2, and PSO3). Healthy skin cells were obtained from three female donors aged 29, 42, and 47 years old who had undergone breast reduction surgery (N = 3 healthy donors, H1, H2, H3). Technical information about the donors is available in Supplementary Table S1.

T lymphocytes were isolated from the blood of a healthy human donor. The blood collection was conducted with approval from the Research Ethics Committee of the Centre Hospitalier Universitaire (CHU) de Québec and in accordance with the principles of the Declaration of Helsinki.

### 2.3 Production of the tissue-engineered human skin substitutes

Tissue-engineered human skin substitutes were prepared according to the methodology detailed in our publication (Ruel et al., 2025). These substitutes consisted of both epidermal and dermal layers, constructed using primary human keratinocytes and fibroblasts. Ascorbic acid was added to the culture medium to stimulate the fibroblast-mediated secretion of extracellular matrix components. For the psoriatic model, dermal sheets were enriched with polarized and activated T lymphocytes, specifically the Th1 and Th17 subsets.

Culture conditions were identical to those described in our work. (Ruel et al., 2025). Dermal sheets, composed solely of fibroblasts, were cultured in Dulbecco’s modified Eagle’s medium (DMEM; ThermoFisher Scientific, Waltham, MA) supplemented with 10% bovine growth serum (FB Essence, Seradigm, Mississauga, ON, Canada), 100 UI/mL penicillin G (MilliporeSigma, Oakville, ON, Canada), and 25 μg/mL gentamicin (Schering, Pointe-Claire, QC, Canada). Once keratinocytes were seeded onto the dermal sheets, the culture medium was replaced with a mixture of three parts DMEM with one part Ham’s F12 (ThermoFisher Scientific), supplemented with 5% Fetal Clone II serum (Galenova, Saint-Hyacinthe, QC, Canada), 100 UI/mL penicillin G (MilliporeSigma), 25 μg/mL gentamicin (Schering), 0.4 μg/mL hydrocortisone (Calbiochem, EMD, Biosciences, Gibbstown, NJ, USA), 5 μg/mL insulin (MilliporeSigma), 10 ng/mL human epidermal growth factor (EGF; Austral Biological, San Ramon, CA, USA), and 10^−10^ M cholera toxin (MP Biomedicals, Montreal, QC, Canada).

The macroscopic photos and the histological cross-sections of the tissue-engineered human skin substitutes corresponding to the samples evaluated in this manuscript are presented in our previous article (Ruel et al., 2025). In these analyses, the microneedle patches loaded with phloretin reduced the epidermal thickness of the psoriatic skin substitutes compared with the untreated psoriatic control, while leaving the dermal thickness unchanged. These effects were even detectable outside the region of the skin substitutes in contact with the patch. A similar antiproliferative response was observed with the microneedle patches loaded with methotrexate, as well as with the systemic-like methotrexate treatment. Interestingly, these effects were specific to the psoriatic skin substitutes: the epidermal thickness of the healthy skin substitutes remained unaffected by any of the treatments, highlighting the specificity of these therapies toward hyperproliferative cells.

The cell culture supernatants were collected at the end of the skin substitute cultures on day 56, which, when applicable, was after 1 week of treatment.

### 2.4 Cytokine array

The Human Cytokine Array C5 (cat. AAH-CYT-5-2, RayBiotech, Peachtree Corners, GA, USA) was employed to quantify 80 cytokines in the cell culture supernatants, following the manufacturer’s instructions. The cell culture supernatants were collected at the end of the treatment week and stored at −80 °C until analysis. Cytokine detection on the membranes was performed using 850 μL of supernatant per condition with the Fusion FX7 (MIB Lab Equipment, Kirkland, QC, Canada). Densitometric quantification of the cytokine spots, based on the integrated density of the spots, was carried out using ImageJ software version 2.14.0. As recommended by the protocol, positive control signals were used to normalize signal responses and enable comparison of results across multiple arrays.

Five experimental conditions were evaluated: the psoriatic control (P CTL), systemic-like methotrexate treatment (MTX SYS), microneedles loaded with methotrexate (MNs MTX), microneedles loaded with phloretin (MNs PHLO), and the healthy control (H CTL). The analyses were performed on supernatants collected from tissue-engineered human skin substitutes generated from primary keratinocytes and fibroblasts derived from three healthy donors for the healthy control (N = 3), and from three donors with plaque psoriasis (N = 3) for all psoriatic conditions, for a total of six biological units.

### 2.5 Quantification of molecules from the supernatants

The secretion levels of specific molecules into the culture medium were assessed using either an Enzyme-Linked Immunosorbent Assay (ELISA) or a bioluminescent assay. These analyses were performed on cell culture supernatants collected at the end of the treatment week and stored at −80 °C until use. To complete the evaluation of the inflammatory response, IL-17A levels were measured using the IL-17A Human ELISA Kit (cat. BMS2017, ThermoFisher Scientific). The metabolic markers insulin and glucose were quantified using the Human Insulin ELISA Kit (cat. KAQ1251, ThermoFisher Scientific) and the Glucose-Glo™ Assay (cat. J6021, Promega Corporation, Fitchburg, WI, USA), respectively. For each assay, a supernatant volume of 50 μL was used for each sample. The same experimental conditions were evaluated: P CTL, MTX SYS, MNs MTX, MNs PHLO, and H CTL. The analyses were performed on supernatants collected from tissue-engineered human skin substitutes generated from primary keratinocytes and fibroblasts derived from three healthy donors for the healthy control (N = 3), and from three donors with plaque psoriasis (N = 3) for all psoriatic conditions, for a total of six biological units. For each condition, supernatants from three independent cultures (n = 3 experimental units) were analyzed per donor group, resulting in a total of nine distinct samples per condition. Each sample supernatant was treated as an experimental unit, and each donor group served as a biological unit, in accordance with the experimental strategies suggested by *Lazic and al* (2018) (Lazic et al., 2018). Absorbance (at 450 nm) and luminescence measurements were performed using the SpectraMax® iD3 microplate reader (Molecular Devices, San Jose, CA, USA). For both glucose and insulin analyses, the concentration of each molecule in the supernatants was quantified. To determine the percentage of uptake by the cells from the culture medium, the ratio of the consumed concentration to the initial concentration in the medium was calculated and multiplied by 100.

### 2.6 Statistics

All statistical analyses were performed using R (v4.4.3) (R Core Team, 2024) in RStudio (v2025.02.28) (Posit Team, 2025) Treatment effects on the various conditions described herein were assessed using either fixed-effects or mixed-effects linear models, depending on whether within-subject effects needed to be accounted for. Mixed-effects models were fitted using the *lme4* package (v1.1.37). (Bates et al., 2015). Model assumptions were verified graphically using the *performance* package (v0.13.0) (Lüdecke et al., 2021). Group-wise marginal means and their 95% confidence intervals (CIs) were estimated using the *ggeffects* package (v2.2.1) (Lüdecke, 2018) and used to generate plots via *ggplot2* (v3.5.2). Effect sizes, reported as model point estimates, and their 95% CIs are provided throughout. Estimates with CIs excluding 0 were interpreted as significant departures from the reference group.

## 3 Results

### 3.1 Anti-inflammatory response induced by microneedles in tissue-engineered human psoriatic skin substitutes

**FIGURE 1:**
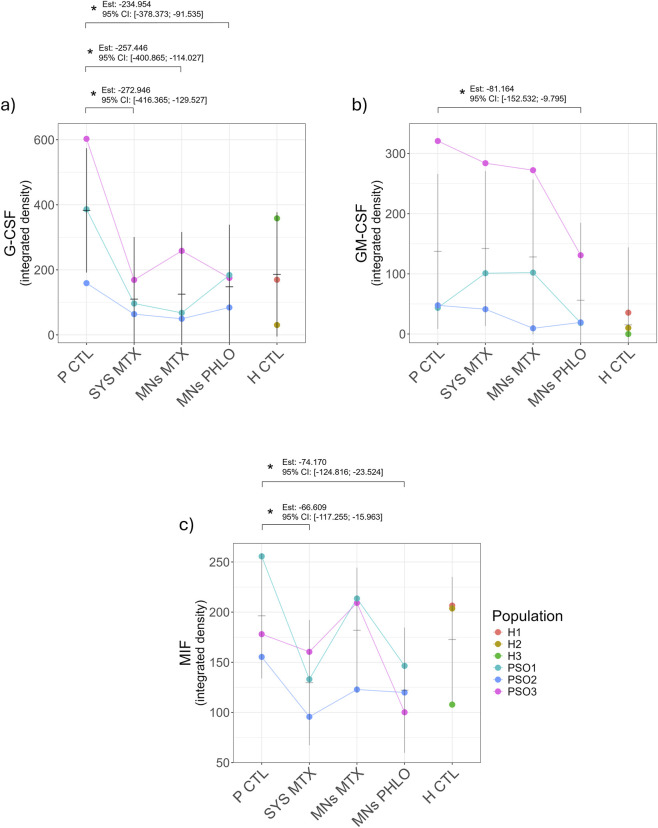
Cytokine array analysis of the inflammatory response. **(a)** Granulocyte colony-stimulating factor (G-CSF), **(b)** granulocyte-macrophage colony-stimulating factor (GM-CSF), **(c)** macrophage migration inhibitory factor (MIF) integrated density levels in the supernatants of tissue-engineered human skin substitutes. Psoriatic and healthy skin substitutes were developed: psoriatic control (P CTL) and healthy control (H CTL). Treatments were applied to psoriatic skin substitutes: systemic-like methotrexate (SYS MTX), methotrexate-loaded microneedle patches (MNs MTX), and phloretin-loaded microneedle patches (MNs PHLO). The analyses were performed on supernatants collected from tissue-engineered human skin substitutes generated from primary keratinocytes and fibroblasts derived from three healthy donors for the healthy control (N = 3; H1, H2, H3), and from three donors with plaque psoriasis for all psoriatic conditions (N = 3; PSO1, PSO2, PSO3). Each donor group is represented by a different colour in the graph. Model-estimated marginal means (horizontal grey ticks) and their 95% confidence intervals (CIs, grey vertical lines) were obtained from a mixed-effects linear model with P CTL as the reference group. The Y-axis is truncated just bellow zero for G-CSF and GM-CSF to reflect biologically plausible values; however, confidence intervals derived from the linear model may extend below zero. Asterisks denote treatment groups for which the estimated difference from P CTL has a 95% CI excluding 0.

Microneedle patches loaded with either methotrexate or phloretin were compared with the systemic-like administration of methotrexate in tissue-engineered human psoriatic skin substitutes. Healthy skin substitutes were also produced to assess whether the treatments could modulate the psoriatic phenotype, inducing a transition toward a healthier skin profile. Assessing the anti-inflammatory activity of the treatments was one of the main objectives of the study. The treatments led to a reduction in pro-inflammatory cytokines, as shown in Figure 1. Among these, granulocyte colony-stimulating factor (G-CSF) was notably affected. Compared with the psoriatic control, G-CSF levels decreased following treatment with systemic-like methotrexate (SYS MTX), methotrexate-loaded microneedle patches (MNs MTX), and phloretin-loaded microneedle patches (MNs PHLO), Figure 1a. The healthy control exhibited an average level of 186.055 integrated density units, indicating a baseline difference of 196.640 units from the psoriatic control (382.695 integrated density units). SYS MTX treatment reduced G-CSF levels to 109.749 units, corresponding to a decrease of 272.946 units compared with the psoriatic control. The 95% CI for this reduction ranged from −416.365 to −129.527 units. MNs MTX resulted in levels of 125.249 units, corresponding to a reduction of 257.446 units (95% CI: −400.865 to −114.027). MNs PHLO treatment yielded levels of 147.741 units, with a decrease of 234.954 units (95% CI: −378.373 to −91.535). Importantly, cytokine levels shifted toward those observed in healthy controls across all donors, and the effects of microneedle-based treatments were comparable to systemic-like methotrexate.

Following treatment with MNs PHLO, the levels of granulocyte-macrophage colony-stimulating factor (GM-CSF) were reduced (Figure 1b). The psoriatic and healthy control levels were 137.364 integrated density units and 15.209 integrated density units, respectively, indicating a difference of 122.155 units between the two controls. The levels of GM-CSF with MNs PHLO were 56.200 integrated density units. This reduction was supported by a 95% CI ranging from −152.532 to −9.795 units, suggesting a significant decrease.

Macrophage migration inhibitory factor (MIF) levels were not substantially different between the control groups, with values of 196.331 integrated density units for the psoriatic control and 172.658 units for the healthy control. However, MIF levels were significantly reduced following treatment with SYS MTX and MNs PHLO (Figure 1c). After SYS MTX treatment, MIF levels decreased to 129.722 units, corresponding to a reduction of 66.609 integrated density units (95% CI: −117.255 to −15.963). Similarly, MNs PHLO treatment resulted in MIF levels of 122.161 units, indicating a reduction of 74.170 integrated density units (95% CI: −124.816 to −23.524).

Supplementary Table S2 presents the mean cytokine levels quantified by the integrated density of array blots. The results of the statistical analysis for Figure 1, performed using a mixed-effects linear model, are provided in Supplementary Table S3. Supplementary Figure S1 shows the membranes from the cytokine array.

**FIGURE 2:**
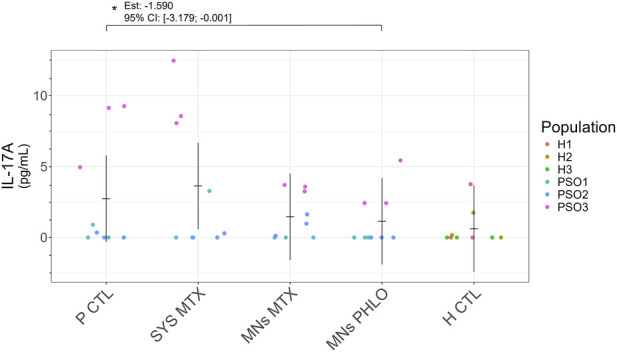
Inflammatory response. Quantification of IL-17A secretion levels (pg/mL) in the supernatants of tissue-engineered human skin substitutes was carried out using ELISA. Psoriatic and healthy skin substitutes were developed: psoriatic control (P CTL) and healthy control (H CTL). Treatments were applied to psoriatic skin substitutes: systemic-like methotrexate (SYS MTX), methotrexate-loaded microneedle patches (MNs MTX), and phloretin-loaded microneedle patches (MNs PHLO). The analyses were performed on supernatants collected from tissue-engineered human skin substitutes generated from primary keratinocytes and fibroblasts derived from three healthy donors for the healthy control (N = 3; H1, H2, H3), and from three donors with plaque psoriasis for all psoriatic conditions (N = 3; PSO1, PSO2, PSO3). The sample supernatants were obtained from three different substitutes (*n* = 3), per condition and per donor group, each cultivated in a separate Petri dish, and resulting in a total of nine distinct samples per condition. Each donor group is represented by a different colour in the graph. Model-estimated marginal means (horizontal grey ticks) and their 95% confidence intervals (grey vertical lines) were obtained from a mixed-effects linear model with P CTL as the reference group. Asterisks denote treatment groups for which the estimated difference from P CTL has a 95% CI excluding 0.

Interleukine-17A secretion was also quantified by ELISA (Figure 2). As expected, the levels in the healthy control were lower than in the psoriatic control. Its expression was reduced following MNs PHLO treatment, with a mean reduction of 1.590 pg/mL. The results of the statistical analysis from Figure 2, performed using a mixed-effects linear model, are provided in Supplementary Table S4.

### 3.2 Alterations in the glucose pathway in psoriasis

**FIGURE 3:**
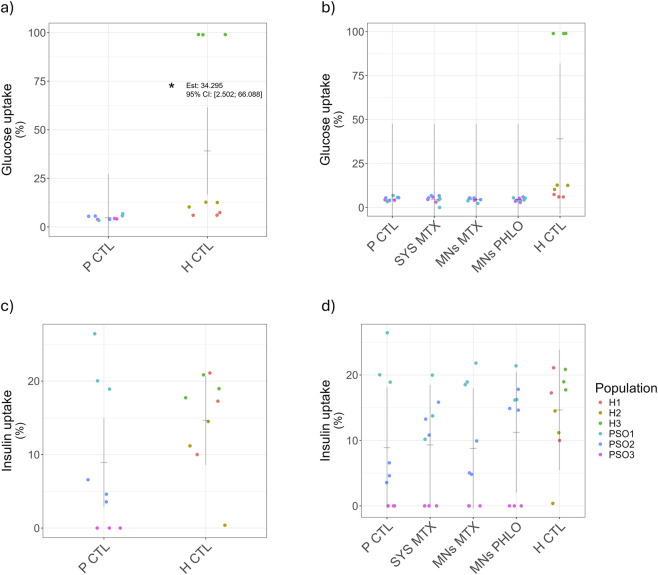
Percentages of **(a,b)** glucose and **(c,d)** insulin uptake from the culture medium by human cells in healthy and psoriatic skin substitute cultures. **(a,c)** Psoriatic skin substitutes (P CTL) were compared with healthy skin substitutes (H CTL). **(b,d)** Treatments were applied to psoriatic skin substitutes: systemic-like methotrexate (SYS MTX), methotrexate-loaded microneedle patches (MNs MTX), and phloretin-loaded microneedle patches (MNs PHLO). The analyses were performed on supernatants collected from tissue-engineered human skin substitutes generated from primary keratinocytes and fibroblasts derived from three healthy donors for the healthy control (N = 3; H1, H2, H3), and from three donors with plaque psoriasis for all psoriatic conditions (N = 3; PSO1, PSO2, PSO3). The sample supernatants were obtained from three different substitutes (*n* = 3), per condition and per donor group, each cultivated in a separate Petri dish, and resulting in a total of nine distinct samples per condition. Each donor group is represented by a different colour in the graph. Model-estimated marginal means (horizontal grey ticks) and their 95% confidence intervals (CIs, grey vertical lines) were obtained from **(a,c)** a fixed-effect linear model and from **(b,d)** a mixed-effects linear model, with P CTL as the reference group. The Y-axis is truncated just below zero in the glucose graphs to reflect biologically plausible values; however, confidence intervals derived from the linear model may extend below zero. The asterisk denotes the treatment group for which the estimated difference from P CTL has a 95% CI excluding 0.

The second main objective of the study was to assess glucose metabolism in the tissue-engineered human psoriatic skin substitutes as compared with healthy ones, and to determine whether the treatments could modulate this metabolic pathway. Psoriatic skin substitutes exhibited lower glucose uptake (4.808%) from the culture medium than the healthy ones (39.103%), representing a difference of 34.295%. This suggests that in the psoriatic model, the cells absorb less glucose (Figure 3a). When comparing the healthy control (H CTL) with the psoriatic control (P CTL), the 95% CI for this reduction ranged from 2.502% to 66.088%. Based on this result, different treatments were assessed to determine whether they could improve the glucose uptake. Neither systemic-like methotrexate (SYS MTX), methotrexate-loaded microneedle patches (MNs MTX), nor phloretin-loaded microneedle patches (MNs PHLO) altered glucose uptake by the cells in the psoriatic skin substitutes (Figure 3b). In addition, the percentage of insulin uptake by the human cells was calculated and found to be lower in culture supernatants of the P CTL (8.904%) than of the H CTL (14.665%), although this difference was not statistically significant (Figure 3c). The treatments were also administered to assess their potential impact on insulin uptake. Most treatments did not affect insulin uptake, except for MNs PHLO, which increased it in one cell population (PSO 2), (Figure 3d). Supplementary Table S5 shows the mean percentages of glucose and insulin uptake from the culture medium by cells in healthy and psoriatic skin substitute cultures. The results of the statistical analysis for Figures 3a,c, performed using a fixed-effects linear model, are presented in Supplementary Table S6. The results of the statistical analysis for Figures 3b,d, performed using a mixed-effects linear model, are shown in Supplementary Table S7.

**FIGURE 4:**
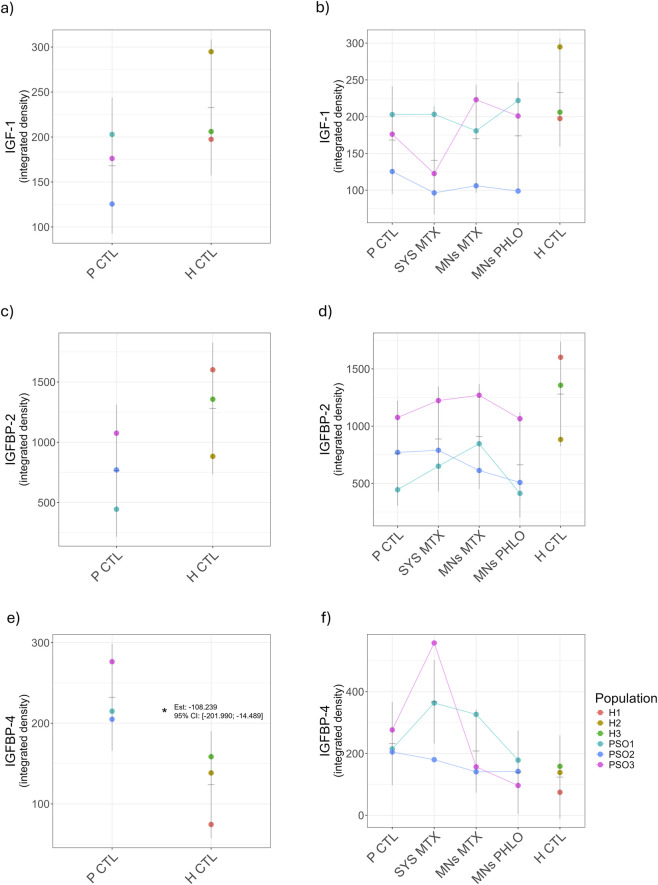
Insulin-like growth factor signalling in healthy and psoriatic human skin models. Protein array on **(a,b)** insulin-like growth factor 1 (IGF-1), **(c,d)** insulin-like growth factor binding protein 2 (IGFBP-2), and **(e,f)** insulin-like growth factor binding protein 4 (IGFBP-4) integrated density levels in supernatants of tissue-engineered human skin substitutes. **(a,c,e)** Psoriatic skin substitutes (P CTL) were compared with healthy skin substitutes (H CTL). **(b,d,f)** Treatments were applied to psoriatic skin substitutes: systemic-like methotrexate (SYS MTX), methotrexate-loaded microneedle patches (MNs MTX), and phloretin-loaded microneedle patches (MNs PHLO). The analyses were performed on supernatants collected from tissue-engineered human skin substitutes generated from primary keratinocytes and fibroblasts derived from three healthy donors for the healthy control (N = 3; H1, H2, H3), and from three donors with plaque psoriasis for all psoriatic conditions (N = 3; PSO1, PSO2, PSO3). Each donor group is represented by a different colour in the graph. Model-estimated marginal means (horizontal grey ticks) and their 95% confidence intervals (CIs, grey vertical lines) were obtained from **(a,c,e)** a fixed-effect linear model and from **(b,d,f)** a mixed-effects linear model, with P CTL as the reference group. The asterisk denotes the treatment group for which the estimated difference from P CTL has a 95% CI excluding 0.

Based on the second main objective of the study to assess glucose metabolism and insulin resistance in the tissue-engineered human psoriatic skin substitutes as compared with healthy ones, insulin-like growth factor signalling was assessed. Insulin-like growth factor 1 (IGF-1) was less abundant in the P CTL than in the H CTL, although the difference was not statistically significant (Figure 4a). The treatments did not affect IGF-1 expression (Figure 4b). Regarding the binding proteins, insulin-like growth factor binding protein 2 (IGFBP-2) was also less present in culture supernatants of the P CTL than those of the H CTL, although the difference was not statistically significant (Figure 4c). The treatments did not impact IGFBP-2 expression (Figure 4d). The most interesting result was the expression of insulin-like growth factor binding protein 4 (IGFBP-4), which was significantly higher in the P CTL than in the H CTL (Figure 4e). P CTL and H CTL yielded levels of 232.052 and 123.813 integrated density units, respectively, with a difference of 108.239 units (95% CI: −201.990 to −14.489). The treatments did not significantly impact IGFBP-4 expression, however, after the MNs PHLO treatment, the levels of IGFBP-4 were closer to those of the H CTL (Figure 4f). MNs PHLO treatment reduced IGFBP-4 levels to 139.007 units. Supplementary Table S8 presents the mean cytokine levels quantified by the integrated density of array blots. The results of the statistical analysis for Figures 4a,c,e, performed using a fixed-effects linear model, are provided in Supplementary Table S9. The results of the statistical analysis for Figures 4b,d,f, performed using a mixed-effects linear model, are shown in Supplementary Table S10.

## 4 Discussion

The first objective of the study was to assess the anti-inflammatory effects of the microneedle patches loaded with either phloretin or methotrexate and to compare these effects with those of systemic-like methotrexate using *in vitro* patient-derived skin models. To carry out this study, tissue-engineered human psoriatic and healthy skin models were used. The levels of multiple cytokines decreased following the treatments in the psoriatic human skin substitutes. First of all, granulocyte colony-stimulating factor (G-CSF) levels were altered following each treatment. G-CSF can be expressed by various cell types, including fibroblasts, endothelial cells and immune cells such as macrophages and monocytes, among others (Cetean et al., 2015). This cytokine also plays a pivotal role in guiding neutrophils to sites of inflammation and enhancing their generation and activation (Katakura et al., 2019). Elevated levels of G-CSF have been observed in the bloodstream of individuals suffering from generalized pustular psoriasis (Wang et al., 2022), a particularly aggressive form of psoriasis marked by widespread pustule formation on the skin, which are densely infiltrated by neutrophils (Marrakchi and Puig, 2022). Intravenous administration of a G-CSF receptor antagonist in healthy subjects who had received subcutaneous G-CSF effectively inhibited neutrophil migration (Gamell et al., 2023). Similarly, in the KC-Tie2 transgenic mouse model exhibiting a psoriatic phenotype, both cutaneous and systemic levels of G-CSF were elevated (Chen et al., 2025). However, no study has been identified in the literature that quantifies G-CSF levels in the skin or serum of patients with plaque psoriasis. In the present study, G-CSF levels in the supernatants of tissue-engineered human psoriatic skin substitutes were found to be twice as high as those in healthy skin substitutes. Following the treatments, there was a 3.5-fold reduction in G-CSF levels with systemic-like methotrexate, a 3.1-fold reduction with methotrexate-loaded microneedle patches, and a 2.6-fold reduction with phloretin-loaded microneedle patches. In the coculture study of human T lymphocytes and psoriatic keratinocytes, phloretin was also found to reduce G-CSF secretion by half (Ruel et al., 2024). Another investigation demonstrated that the exposure of peripheral blood mononuclear cells to phloretin under LPS-induced inflammatory conditions led to a marked decrease in G-CSF levels (Fordham et al., 2014). Regarding methotrexate, its coadministration with G-CSF to healthy neutrophils cultured in a chemotaxis chamber inhibited their migration and reduced superoxide production as compared with the supplementation of G-CSF alone in neutrophils (Okuda et al., 1996). The levels of G-CSF were not measured after the administration of methotrexate; however, the findings demonstrated the anti-inflammatory impact of methotrexate on neutrophils.

In addition, the administration of phloretin-loaded microneedle patches reduced granulocyte-macrophage colony-stimulating factor (GM-CSF) production levels in the psoriatic human skin substitutes. This treatment exhibited superior efficacy than systemic-like methotrexate. At baseline, GM-CSF levels were particularly elevated in the psoriatic control compared with the healthy control. In another study, GM-CSF was also secreted at higher levels by human psoriatic keratinocytes compared with healthy ones and by cocultures of T cells and human psoriatic keratinocytes compared with healthy cocultures (Martin et al., 2012). GM-CSF is produced by various activated immune and non-immune cells, including T lymphocytes, macrophages, fibroblasts, keratinocytes, and endothelial cells (Al-Mossawi et al., 2017; Takematsu and Tagami, 1990). This cytokine plays a key role in promoting inflammatory responses (Hamilton and Anderson, 2004; Lotfi et al., 2019). Notably, GM-CSF enhanced the production of CC motif chemokine ligand 17 (CCL17), which attracts several immune cells, including macrophages, Th2, Th1 and regulatory T (Treg) cells, as well as monocytes, basophils, eosinophils, and dendritic cells (Achuthan et al., 2016; Girkin, 2022; Gilet et al., 2009). Elevated levels of GM-CSF have been detected in the plasma of individuals with moderate to severe psoriasis compared with healthy controls (Lecewicz-Torun et al., 2001). Its overexpression has also been quantified in psoriatic skin lesions, including those associated with erythrodermic psoriasis, pustular forms, palmoplantar pustulosis, but particularly with plaque psoriasis (Takematsu and Tagami, 1990). Moreover, phloretin treatment of LPS-stimulated peripheral blood mononuclear cells resulted in a non-significant reduction in GM-CSF levels (Fordham et al., 2014). In the coculture of human T lymphocytes and psoriatic keratinocytes, phloretin completely inhibited GM-CSF production, in contrast to systemic-like methotrexate (Ruel et al., 2024). These differences between the results with phloretin and methotrexate can be explain by their different mechanisms of action. Methotrexate is a folic acid antagonist, which accounts for its strong antiproliferative activity, and also exerts immunomodulatory effects through systemic regulation of immune cell proliferation and activation (Maxwell et al., 1990; Furiati et al., 2019). Indirectly, methotrexate can modulate inflammatory activity, notably through pathways such as NF-κB signaling (Spurlock et al., 2015). On the other hand, phloretin exhibits potential therapeutic effects for the treatment of multiple pathologies, particularly inflammatory diseases and diabetes. Phloretin acts directly on key anti-inflammatory pathways (e.g., NF-κB, MAPK, and Nrf2) (Chang et al., 2012; Hytti et al., 2023). Regarding its antidiabetic activity, this polyphenolic derivative acts as an inhibitor of glucose transporter type 2 (GLUT2) and sodium–glucose cotransporter 1 (SGLT-1) (Williamson, 2022; Bergling et al., 2022; Shen et al., 2020; Shelke et al., 2024).

The integrated density of macrophage migration inhibitory factor (MIF) secretion in the supernatants of psoriatic human skin substitutes showed a decrease with the administration of both systemic-like methotrexate and phloretin-loaded microneedle patches. MIF was first discovered as a lymphokine, which is a molecule secreted by lymphocytes. In this context, MIF is released by Th1, Th2, Th17 and B cells. However, this cytokine is also expressed by many other immune cells, such as monocytes, macrophages, dendritic cells, neutrophils, eosinophils, mast cells, and basophils. MIF also attracts macrophages, dendritic cells, and T and B lymphocytes through its affinity for their CD74 receptors (Calandra and Roger, 2003; Stošić-Grujičić et al., 2020). In addition, keratinocytes exhibit higher MIF expression than fibroblasts, which also express MIF but at approximately half the level (Brocks et al., 2017). MIF has been shown to activate extracellular signal-regulated kinase 1 and 2 (ERK1 and ERK2), which are key components of the mitogen-activated protein kinase (MAPK) signalling pathway (Calandra and Roger, 2003). This pathway is overactivated in psoriasis, with increased expression of ERK1/2 and MAPK p38 in psoriatic lesional skin than in non-lesional skin (Johansen et al., 2005). In the lesional skin of patients with psoriasis, MIF was found to be overexpressed in the keratinocytes of the suprabasal layers of the epidermis, mostly in the spinous layer (Steinhoff et al., 1999). MIF is also overexpressed in the serum of patients with psoriasis vulgaris compared with healthy controls. Higher MIF concentrations were also correlated with higher PASI scores (Shimizu et al., 2001). In lymph node cells from mice, recombinant MIF increased interleukin-17 (IL-17) expression levels. Conversely, IL-17 secretion was reduced in MIF knockout mice (Stojanović et al., 2009). Additionally, phloretin inhibits the enzymatic activity of MIF phenylpyruvate tautomerase, involved in MIF signalling (Molnar and Garai, 2005). These findings could explain why MIF levels were reduced following the treatment with phloretin-loaded microneedle patches. Another study explored the effect of methotrexate in patients with rheumatoid arthritis. After the treatment, the synovial fluid contained less MIF (Morand et al., 2002). These data are supported by the interaction between the carboxyl group moiety of methotrexate and the active site of MIF, as revealed by X-ray crystal structural analysis (Fukushima et al., 2021). Our results are consistent with the literature: systemic-like methotrexate reduced the expression of MIF in the psoriatic skin substitute supernatants.

Importantly, interleukin-17A (IL-17A) is involved in the main inflammatory pathway of psoriasis. This cytokine is secreted by Th17 cells (Ogawa et al., 2018). IL-17A enhances keratinocyte proliferation, which stimulates the production of other cytokines, including interleukin-6 (IL-6), interleukin-8 (IL-8), G-CSF, and GM-CSF, among others, and chemokines, such as CC motif chemokine ligand 20 (CCL20) (Mosca et al., 2021). The chemokines attract neutrophils to the site of inflammation (Ogawa et al., 2018). IL-17 was highly expressed in the serum of patients with plaque psoriasis compared with healthy volunteers (Nassar et al., 2022). In the present study, IL-17A expression was reduced following the treatment with phloretin-loaded microneedle patches, reaching levels comparable to those observed in the healthy control. In the same way, phloretin administered in the culture medium of cocultures of human T lymphocytes and psoriatic keratinocytes reduced IL-17A levels to values comparable to those of the healthy control. The reduction in IL-17A production levels using phloretin was also greater than that observed with methotrexate (Ruel et al., 2024). In the present study with the psoriatic human skin substitutes, there was no statistically significant change in IL-17A levels upon treatment with the systemic-like administration of methotrexate. This confirmed that phloretin may exert a stronger effect than methotrexate on the main axis pathway of psoriasis.

To conclude for this objective, which focused on assessing the anti-inflammatory activity of treatments with phloretin-loaded microneedle patches, methotrexate-loaded microneedle patches and systemic-like methotrexate, phloretin appears to be a particularly interesting compound for loading into microneedle patches for the treatment of psoriasis. Its anti-inflammatory effects were demonstrated in tissue-engineered human psoriatic skin substitutes, developed using fibroblasts and keratinocytes from patients with plaque psoriasis, and enriched with human T cells, with reductions observed in the levels of G-CSF, GM-CSF, MIF and IL-17A. Systemic-like treatment with methotrexate reduced G-CSF and MIF levels, whereas methotrexate-loaded microneedle patches only decreased G-CSF levels.

Furthermore, the second objective aimed to assess glucose metabolism and insulin resistance, both of which are associated with type 2 diabetes, a comorbid condition frequently linked to psoriasis, using the tissue-engineered human psoriatic and healthy skin models, prior to evaluating the microneedle treatments. Glucose uptake by cells in the psoriatic human skin substitutes was lower, approximately eight times less, than in cells from the healthy skin substitutes. This reflects reduced glucose absorption by the cells from donors with plaque psoriasis under inflammatory conditions, characterized by T lymphocyte infiltration. Glucose consumption in psoriatic skin substitutes also remained minimal overall, with only about 4.8% of the available glucose in the culture medium being utilized, compared with approximately 39.1% consumed by cells from the healthy skin substitutes. The psoriatic condition was associated with elevated glucose concentrations in the cell culture supernatants, suggesting altered metabolic activity or impaired glucose uptake. In patients with psoriasis, these data may reflect raised blood glucose concentrations.

Elevated blood glucose levels have also been shown to exert pro-inflammatory effects (Avtanski and Poretsky, 2023). In our cultures, more specifically in the psoriatic skin substitute cultures, the high concentration of glucose in the medium, which is not as well absorbed as in the healthy condition, can influence and exacerbate the inflammatory conditions. Notably, findings from a previous study indicate that elevated glucose concentrations in cultures of human pancreatic β-cells, even from non-diabetic donors, increase interleukin-1 β (IL-1β) expression, which alters insulin secretion. In detail, the upregulation of IL-1β activates nuclear factor kappa B (NF-κB) and promotes the expression of Fas ligand, also known as FS7-associated cell surface antigen, CD95, APO-1 or TNFRSF6, a protein that, upon binding to its receptor, enhances apoptotic signalling. These molecular events lead to DNA fragmentation and ultimately impair pancreatic β-cell function by reducing insulin secretion (Maedler et al., 2002). Moreover, Il-17A appears to be an important cytokine upregulated in type 2 diabetes and that contributes to the chronic inflammation (Abdel-Moneim et al., 2018). This cytokine also contributes to the chronic inflammation in psoriasis (Ogawa et al., 2018). The underlying mechanism may involve the induction of several other pro-inflammatory cytokines, such as IL-1β, IL-6, and, in particular, tumor necrosis factor alpha (TNF-α), which are known to promote insulin resistance (Abdel-Moneim et al., 2018; Wieser et al., 2013; McGinley et al., 2020; Chen and Shao, 2016; Hotamisligil, 2006). Mechanistically, TNF-α participates in a serine phosphorylation of the insulin receptor substrate-1 (IRS-1), which impairs its ability to effectively participate in insulin signalling. This disruption hinders the intracellular signalling pathway PI3K/Akt, thereby contributing to insulin resistance (Abdel-Moneim et al., 2018; Hotamisligil, 2006). Interestingly, anti-TNF-α therapy has been associated with improved insulin sensitivity in individuals with rheumatoid arthritis (Hotamisligil, 2006; Lim et al., 2024). In the tissue-engineered human psoriatic skin model, the presence of activated T cells, which mediate IL-17A production (Rioux et al., 2021), influenced the production of several inflammatory markers and likely affected the insulin resistance pathway, and thereby glucose uptake. Additionally, in the present study the higher secretion of GM-CSF by the psoriatic skin substitutes could contribute to lower insulin sensitivity. GM-CSF knockout mice on a high-fat diet exhibited increased insulin sensitivity and enhanced glycolysis compared with wild-type mice. These findings are explained by a general reduction in inflammation, with decreased macrophage infiltration in mesenteric fat and lower levels of inflammatory markers such as IL-1β, TNF-α, and macrophage inflammatory protein-1 alpha (MIP-1α) (Kim et al., 2008). Another cytokine analyzed in our study, G-CSF, has been reported in the literature to induce insulin desensitization. For example, in human skeletal muscle cells differentiated *in vitro* (myotubes) and adipocytes, both derived from insulin-resistant donors and supplemented with G-CSF, insulin-induced Akt phosphorylation was reduced (Ordelheide et al., 2016). G-CSF production in the psoriatic cultures was elevated, and this cytokine could have participated in a reduction in insulin sensitivity. In contrast, MIF stands out as a particularly unique cytokine. On the one hand, MIF plays a role in insulin release in pancreatic β-cells. Under normal conditions, these cells secrete MIF as well as other polypeptides, and MIF stimulates insulin secretion. On the other hand, in inflammatory diseases elevated levels of MIF drive inflammation and trigger a downstream inflammatory cascade that contributes to insulin resistance (Stošić-Grujičić et al., 2020). However, it can be difficult to determine which level of MIF would be sufficient to induce insulin resistance.

Given the observed dysregulation of glucose consumption by human cells in the psoriatic skin substitutes, in the first place, insulin uptake was analyzed. Unsurprisingly, insulin uptake by cells was lower in the psoriatic skin substitutes than in the healthy skin substitutes, respectively 8.904% and 14.665%, without, however, detecting significant differences. This is equivalent to having higher insulin levels in the psoriatic condition supernatants. In line with a previous study, increased insulin expression has been observed in the blood of patients with psoriasis alone, as well as in those with both psoriasis and diabetes compared with healthy controls (Brazzelli et al., 2021). In another clinical study, patients with psoriasis and no history of cardiovascular disease had elevated insulin levels (Janusz et al., 2010). In humans, when insulin signalling is impaired, pancreatic β-cells increase insulin production (Dludla et al., 2023), which may explain why pronounced differences are more difficult to observe in the *in vitro* skin model, where insulin supplementation is uniform and differences can only be detected through consumption.

Understanding insulin signalling requires acknowledging the structural and functional similarities between insulin and insulin-like growth factor 1 (IGF-1). IGF-1 is a peptide hormone with a similar structure to insulin. It mostly binds to the IGF-1 receptor (IGF-1R), but to a lesser extent, to the insulin receptor (IR) as well. A hybrid receptor, formed by the combination of IGF-1 and IR, exhibits high affinity for IGF-1 and lower affinity for insulin. When IGF-1 binds to its receptor IGF-1R, intracellular signalling pathways such as PI3K/Akt are activated, pathways that are also stimulated by insulin. These pathways enhance the intracellular absorption of glucose and play key roles in regulating metabolism, cell growth, and differentiation. More specifically, when IGF-1 binds to IGF-1R, located on the cell membrane, Insulin Receptor Substrates 1 and 2 (IRS-1/2) are activated, similarly to the mechanism triggered by insulin. IRS proteins then activate Phosphoinositide 3-Kinase (PI3K), which catalyzes the conversion of PIP2 (phosphatidylinositol 4,5-bisphosphate) into PIP3 (phosphatidylinositol 3,4,5-trisphosphate). PIP3 activates Protein Kinase B (PKB), also known as serine/threonine kinase (AKT). AKT promotes the translocation of Glucose Transporter Type 4 (GLUT4) vesicles to the cell membrane. Once at the membrane, GLUT4 facilitates the entry of glucose into the cell (Haywood et al., 2019; Acosta-Martinez and Cabail, 2022).

IGF-1 can be secreted by fibroblasts and internalized by keratinocytes via their IGF-1 receptors, which stimulates their proliferation (Lewis et al., 2010). In psoriasis, the epidermal hyperplasia resulting from keratinocyte hyperproliferation is accentuated (Ogawa et al., 2018). In type 2 diabetes, IGF-1 plays a different role and regulates insulin uptake. However, how does it affect patients with psoriasis and type 2 diabetes? IGF-1 expression in psoriasis is controversial. In the skin of patients with plaque psoriasis, IGF-1 was found to be upregulated (El-Komy et al., 2011), in particular in lesional skin (Hodak et al., 1996). IGF-1 receptors are highly expressed on the keratinocytes of psoriatic skin compared with healthy skin (Krane et al., 1992). However, the expression of IGF-1 was lower in the blood of patients with moderate to severe psoriasis and without a metabolic syndrome (no treatment affecting the metabolism prior to 6 months) than in healthy subjects (Savastano et al., 2011). The quantification of IGF-1 in cell culture supernatants is more comparable with the quantification of IGF-1 in the blood of patients. Blood and culture supernatants are both fluids that enable the exchanges of secreted and absorbed molecules by cells. Our findings are consistent with the results from the study analyzing IGF-1 concentrations in the blood of patients; IGF-1 levels in the supernatants of the psoriatic skin substitutes were lower. Also, in a clinical study involving untreated patients with moderate to mild psoriasis and healthy volunteers, no difference in IGF-1 levels was observed between the two groups (Björntorp et al., 1997). Another study showed that patients with obesity or insulin resistance had lower IGF-1 levels in their serum. Conversely, patients with poorly controlled type 2 diabetes had higher IGF-1 levels (AbdlWhab et al., 2024). In a cohort of 3,354 subjects, high and low IGF-1 levels were correlated with insulin resistance (elevated and low HOMA-IR values, Homeostatic Model Assessment for Insulin Resistance) (Friedrich et al., 2012). HOMA1-R values are calculated by multiplying fasting insulin (µU/mL) by fasting glucose (mmol/L), and dividing the result by 22.5 (Matthews et al., 1985; Minh et al., 2021). This has been explained by the fact that when an elevated blood glucose is maintained, as in type 2 diabetes, IGF-1 levels diminish incrementally (Clauson et al., 1998). In the present study, the psoriatic human skin substitutes appear to exhibit features of insulin resistance, as evidenced by a significant reduction in glucose uptake. The lower IGF-1 levels in the supernatants, along with the lower insulin uptake, currently support that point.

**FIGURE 5:**
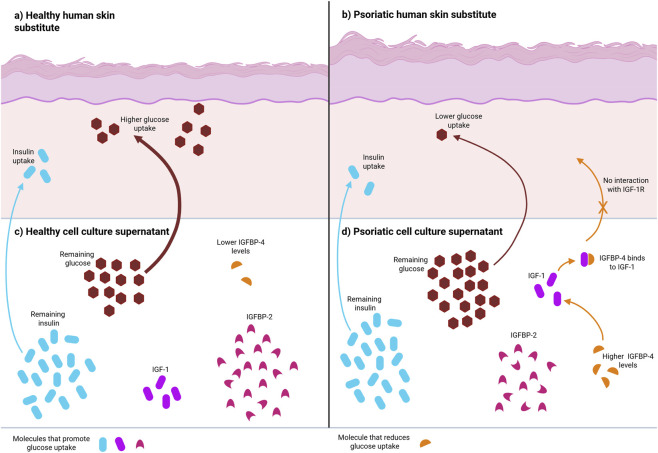
Glucose and insulin uptake, as well as insulin-like growth factor signalling (IGF) in **(a)** the healthy human skin substitutes compared with **(b)** the psoriatic human skin substitutes. **(c)** Molecules remaining or secreted in the **(c)** healthy cell culture supernatant or **(d)** psoriatic cell culture supernatant are represented. The cells from the psoriatic skin substitutes consume approximately eight times less glucose than those from the healthy skin substitutes. Higher levels of insulin-like growth factor binding protein 4 (IGFBP-4) in the psoriatic cell culture supernatants may explain the reduced glucose uptake. Insulin, insulin-like growth factor 1 (IGF-1) and insulin-like growth factor binding protein 2 (IGFBP-2) promote glucose uptake.

Furthermore, the interaction of IGF-1 with binding proteins IGFBP-1 through IGFBP-6 modulates its biological effects. Indeed, these proteins bind IGF-1 and regulate its availability to receptors. Among these, insulin-like growth factor binding protein 4 (IGFBP-4) reduces the interaction of IGF-1 with IGF-1R, thereby leading to decreased stimulation of glucose uptake pathways (Haywood et al., 2019). As a result, higher levels of IGFBP-4 may reduce glucose absorption by cells, leaving more glucose in the extracellular environment or culture medium. Furthermore, in knockout mice lacking IGFBP-3, -4, and -5, the number of pancreatic β-cells was increased. Glucose tolerance was also improved: following glucose injection, insulin levels were significantly higher than in wild-type mice. Although IGF-I levels in plasma were lower, the percentage of increase in IGF-I secretion after 30 min of injection was higher than in wild-type mice. Circulating glucose levels were reduced, reflecting enhanced glucose clearance and improved cellular uptake. In contrast, blocking either one of the molecules IGFBP-3, -4, or -5 individually did not significantly affect metabolism (Ning et al., 2006). Based on the experimental observations in the present study, IGFBP-4 emerged as one of the most abundant IGF-binding proteins in the cell culture medium, preceded by IGFBP-2. This finding is consistent with previous studies involving cultured human adipose tissue (Gude et al., 2012), suggesting a broader role for IGFBP-4 in modulating IGF-1 activity and glucose metabolism. IGFBP-4 levels were elevated in the supernatants of the psoriatic human skin substitutes compared with healthy controls, which could explain the lower glucose uptake in the disease model. Conversely, IGFBP-2 increased glucose uptake in adipocytes, independently of insulin and IGF-1 receptor activity, by activating various pathways that promote GLUT4 translocation (Assefa et al., 2017). In the present study, IGFBP-2 expression in the supernatants of the psoriatic human skin substitutes was at lower levels than that of healthy substitutes. This result would be consistent with the previously described study and may reflect increased glucose uptake in healthy keratinocytes compared with psoriatic ones. In diabetic patients, IGFBP-2 was found at lower levels (Boughanem et al., 2021). In other studies, no difference in IGFBP-3 expression was observed between senescent psoriatic keratinocytes and healthy ones (Mercurio et al., 2020), nor between psoriatic and healthy fibroblasts (Miura et al., 2000). IGFBP-3 was also produced at lower levels than IGFBP-2 (Mercurio et al., 2020), which is consistent with our findings. This prompts further investigation into the roles of IGFBP-4 and IGFBP-2 in the psoriatic 3D model, particularly regarding their potential involvement in psoriasis pathophysiology and glucose metabolism. Figure 5 summarizes the main imbalances observed in glucose metabolism between the healthy human skin substitutes and the psoriatic human skin substitutes.

This study allowed the evaluation of insulin and glucose uptake by primary human psoriatic cells. Specifically, it aimed to determine whether cells from the psoriatic human skin substitutes exhibit dysregulated consumption of these molecules from the culture medium compared with cells from the healthy skin substitutes. This psoriatic model could potentially reflect metabolic imbalances similar to those observed in type 2 diabetes, where glucose uptake is impaired. In type 1 diabetes, pancreatic β-cells are destroyed by the immune system, leading to insufficient or absent insulin production (Holt and Flyvbjerg, 2024). The experimental conditions used for the culture of the skin substitutes were not suitable for evaluating the dysregulation associated with type 1 diabetes because insulin was uniformly supplemented across all cultures. Consequently, the psoriatic patient-derived skin model proved to be a valuable platform for investigating the type 2 diabetes comorbidity in the context of psoriasis.

Other models in the literature assessed the comorbid presence of metabolic dysfunctions in psoriatic conditions. In an imiquimod (IMQ)-induced psoriatic mouse model, glucose tolerance improved, with a decrease in blood glucose, due to the increased pancreatic β-cell proliferation and plasma insulin levels (Evans et al., 2020). However, reduced blood glucose is not characteristic of human type 2 diabetes. While it is interesting to observe metabolic differences in this model, the findings confirm that an induced psoriatic phenotype alone is insufficient to model the comorbid presence of type 2 diabetes in a mouse model. Type 2 diabetes is not innate in mice (LeRoith and Gavrilova, 2006). To induce a type 2 diabetes-like state in a mouse model, streptozotocin can be administered at a moderate dose to partially destroy pancreatic β-cells and increase blood glucose levels, especially when combined with a high-fat and high-sugar diet (Shen et al., 2017; Mazo et al., 2016; Sharma et al., 2023). The administration of streptozotocin alone at a higher dose primarily induces type 1 diabetes rather than insulin resistance, as observed in type 2 diabetes (Sharma et al., 2023; Akbarzadeh et al., 2007). Regarding *in vitro* models, a human induced pluripotent stem cell (iPSC) model for psoriasis was developed using keratinocytes from patients with both psoriasis and insulin resistance. Healthy controls, using keratinocytes from healthy donors, were included. These iPSCs were differentiated into mature keratinocytes. Dysregulated genes associated with glucose transporters (*GLUT10* and *GLUT14)* and insulin receptor substrate 2 (*IRS2*) were identified in the psoriatic model (Ali et al., 2020). However, this culture lacks epidermal and dermal components. In addition, the inflammatory profile associated with psoriasis and insulin resistance is incomplete due to the absence of immune cells.

In the psoriatic human skin substitutes, glucose and insulin uptake by the cells were lower than in the healthy human skin substitutes, corresponding to a type 2 diabetic phenotype. To our knowledge, no other tissue-engineered 3D models have been reported in the literature that include both epidermal and dermal components with cells derived from patients with psoriasis to assess insulin resistance. This psoriatic human skin substitute could serve as a valuable model for investigating insulin resistance in psoriasis and type 2 diabetes comorbidities.

Additionally, following the administration of phloretin-loaded microneedle patches, insulin uptake was slightly increased, likely due to the effects observed in one psoriatic donor (PSO2), although no statistically significant changes were detected. A larger number of donors would have been necessary to observe significant differences between the evaluated treatments and the psoriatic control. Increasing the number of donors would enable a more comprehensive analysis across a larger cohort, thereby helping to determine whether the observed effects are donor-specific or representative of a consistent, universal trend. Moreover, comparing donors with distinct metabolic profiles, such as individuals with psoriasis alone and others with psoriasis associated with diagnosed type 2 diabetes or insulin resistance, would further strengthen the personalized medicine potential of this therapy. Such stratification would enable a better understanding of how baseline metabolic status influences responsiveness to this microneedle-based therapy and could ultimately support more tailored therapeutic approaches, helping to determine whether this therapy could be more effective in patients with more pronounced baseline metabolic imbalances. Besides, the treatments did not affect glucose uptake or IGF-1 levels. Interestingly, treatment with phloretin-loaded microneedle patches led to a reduction in IGFBP-4 expression, bringing its levels closer to those observed in the healthy control, although no statistically significant changes were detected. Thus, phloretin could modulate IGFBP-4 expression. No significant changes in IGFBP-2 levels were observed following the treatments. Considering the modulatory effects of various cytokines and chemokines on insulin resistance discussed above, the anti-inflammatory properties of phloretin-loaded microneedle patches may have the potential to influence glucose uptake. While microneedles loaded with phloretin markedly improved the inflammatory profile in the tissue-engineered human psoriatic skin model, the lack of a concomitant significant improvement in glucose uptake within the same 1-week period suggests that the resolution of insulin resistance may require a longer timeframe. Indeed, inflammatory and metabolic pathways, although interconnected (Abramczyk et al., 2020), may not respond with the same kinetics. Improvements in inflammatory signaling may precede measurable changes in glucose metabolism, especially in a complex, tissue-engineered model. Extending the treatment duration may therefore be necessary to observe a more pronounced effect on insulin resistance. In particular, treatment periods beyond 1 week (e.g., up to 2 weeks) could further enhance the therapeutic benefits and improve the statistical significance of metabolic outcomes. This extended duration could also allow microneedles to release a greater amount of their therapeutic payload. Moreover, our previous study showed that these microneedles made of PLGA/PLA and loaded with phloretin are capable of biodegrading over a period of at least 2 weeks, thereby potentially providing sustained therapeutic benefits throughout this time frame (Ruel et al., 2025). Also, increasing the loading dose of phloretin in the microneedles or combining phloretin with another therapeutic molecule within the microneedles could enhance the effectiveness of the treatment by simultaneously targeting both psoriasis and insulin resistance. Nonetheless, the drug-loading capacity of microneedles is limited to ensure their structural and mechanical stability. Phloretin administered to streptozotocin-induced diabetic rats reduced blood glucose and plasma insulin levels, as well as HOMA-IR values. Similarly, in a myoblast cell line, glucose consumption increased after phloretin treatment, with higher doses leading to greater glucose uptake. These results suggest that phloretin enhances sensitivity to insulin and holds potential for the treatment of type 2 diabetes (Shen et al., 2017).

## 5 Conclusion

This tissue-engineered human psoriatic skin model exhibits features characteristic of type 2 diabetes, including downregulated glucose and insulin uptake, as well as impaired insulin-like growth factor signalling. It represents the first human model that includes both epidermal and dermal components with cells derived from patients with psoriasis to simultaneously reflect the inflammatory profile of psoriasis and insulin resistance. As such, it offers a valuable platform for investigating the comorbidity between psoriasis and type 2 diabetes, and for evaluating potential therapeutic strategies. Notably, phloretin-loaded microneedle patches were assessed as a treatment and demonstrated anti-inflammatory effects by lowering cytokine levels involved in both conditions. These patches were more effective than both systemic-like methotrexate and methotrexate-loaded microneedle patches. Moreover, they demonstrated further potential by enhancing insulin uptake and increasing the levels of insulin-like growth factor binding protein 4 (IGFBP-4) in the supernatants, although the effects were not statistically significant. However, extending the treatment duration beyond 1 week or combining it with another molecule that enhances insulin sensitivity could improve the efficacy of this therapy by targeting both psoriasis and type 2 diabetes.

## Data Availability

The original contributions presented in the study are included in the article/Supplementary Material, further inquiries can be directed to the corresponding author.

## References

[B1] Abdel-Moneim A. Bakery H. H. Allam G. (2018). The potential pathogenic role of IL-17/Th17 cells in both type 1 and type 2 diabetes mellitus. Biomed. and Pharmacother. 101, 287–292. 10.1016/j.biopha.2018.02.103 29499402

[B2] AbdlWhab H. M. Al-Saffar A. Mahdi O. A. Alameri R. B. (2024). The impact of insulin resistance and glycaemic control on insulin-like growth factor-1 in patients with type 2 diabetes: a cross-sectional study. Clin. Diabetes Endocrinol. 10 (1), 36. 10.1186/s40842-024-00202-8 39578883 PMC11585245

[B3] Abramczyk R. Queller J. N. Rachfal A. W. Schwartz S. S. (2020). Diabetes and psoriasis: different sides of the same prism. Diabetes Metab. Syndr. Obes. 13, 3571–3577. 10.2147/DMSO.S273147 33116708 PMC7548229

[B4] Achuthan A. Cook A. D. Lee M. C. Saleh R. Khiew H. W. Chang M. W. (2016). Granulocyte macrophage colony-stimulating factor induces CCL17 production *via* IRF4 to mediate inflammation. J. Clin. Invest. 126 (9), 3453–3466. 10.1172/JCI87828 27525438 PMC5004969

[B5] Acosta-Martinez M. Cabail M. Z. (2022). The PI3K/Akt pathway in meta-inflammation. Int. J. Mol. Sci. 23 (23), 15330. 10.3390/ijms232315330 36499659 PMC9740745

[B6] Akbarzadeh A. Norouzian D. Mehrabi M. R. Jamshidi S. Farhangi A. Verdi A. A. (2007). Induction of diabetes by streptozotocin in rats. Indian J. Clin. Biochem. 22 (2), 60–64. 10.1007/BF02913315 23105684 PMC3453807

[B7] Al-Mossawi M. H. Ridley A. Chen L. Y. de Wit J. Bowness P. (2017). Role of lymphocytes producing GM-CSF in human spondyloarthritis. Lancet 389, 21. 10.1016/s0140-6736(17)30417-8 28091368

[B8] Ali G. Elsayed A. K. Nandakumar M. Bashir M. Younis I. Abu Aqel Y. (2020). Keratinocytes derived from patient-specific induced pluripotent stem cells recapitulate the genetic signature of psoriasis disease. Stem Cells Dev. 29 (7), 383–400. 10.1089/scd.2019.0150 31996098 PMC7153648

[B9] Armstrong A. W. Harskamp C. T. Armstrong E. J. (2013). Psoriasis and the risk of diabetes mellitus: a systematic review and meta-analysis. JAMA Dermatol. 149 (1), 84–91. 10.1001/2013.jamadermatol.406 23407990

[B10] Assefa B. Mahmoud A. M. Pfeiffer A. F. H. Birkenfeld A. L. Spranger J. Arafat A. M. (2017). Insulin-like growth factor (IGF) binding Protein-2, independently of IGF-1, induces GLUT-4 translocation and glucose uptake in 3T3-L1 adipocytes. Oxid. Med. Cell. Longev. 2017, 3035184. 10.1155/2017/3035184 29422987 PMC5750484

[B11] Avtanski D. Poretsky L. (2023). Obesity, Diabetes and Inflammation: Molecular Mechanisms and Clinical Management. Cham: Springer.

[B12] Bates D. Mächler M. Bolker B. Walker S. (2015). Fitting linear mixed-effects models using lme4. J. Stat. Softw. 67 (1), 1–48. 10.18637/jss.v067.i01

[B13] Bélanger A. Grenier A. Simard F. Gendreau I. Pichette A. Legault J. (2019). Dihydrochalcone derivatives from Populus balsamifera L. buds for the treatment of psoriasis. Int. J. Mol. Sci. 21 (1), 1–15. 10.3390/ijms21010256 31905943 PMC6981943

[B14] Bergling K. Martus G. Öberg C. M. (2022). Phloretin improves ultrafiltration and reduces glucose absorption during peritoneal dialysis in rats. J. Am. Soc. Nephrol. 33 (10), 1857–1863. 10.1681/asn.2022040474 35985816 PMC9528341

[B15] Bi D. Qu F. Xiao W. Wu J. Liu P. Du H. (2023). Reactive oxygen species-responsive gel-based microneedle patches for prolonged and intelligent psoriasis management. ACS Nano 17 (5), 4346–4357. 10.1021/acsnano.2c08979 36847798

[B16] Björntorp E. Wickelgren R. Bjarnason R. Swanbeck G. Carlsson L. M. Lindahl A. (1997). No evidence for involvement of the growth hormone/insulin-like growth factor-1 axis in psoriasis. J. Invest. Dermatol 109 (5), 661–665. 10.1111/1523-1747.ep12337699 9347796

[B17] Bouchard C. Grenier A. Cardinal S. Bélanger S. Voyer N. Pouliot R. (2022). Antipsoriatic potential of quebecol and its derivatives. Pharmaceutics 14 (6), 1129. 10.3390/pharmaceutics14061129 35745702 PMC9227144

[B18] Boucher J. Kleinridders A. Kahn C. R. (2014). Insulin receptor signaling in normal and insulin-resistant states. Cold Spring Harb. Perspect. Biol. 6 (1), 1–23. 10.1101/cshperspect.a009191 24384568 PMC3941218

[B19] Boughanem H. Yubero-Serrano E. M. López-Miranda J. Tinahones F. J. Macias-Gonzalez M. (2021). Potential role of insulin growth-factor-binding protein 2 as therapeutic target for obesity-related insulin resistance. Int. J. Mol. Sci. 22 (3), 1133. 10.3390/ijms22031133 33498859 PMC7865532

[B20] Brazzelli V. Maffioli P. Bolcato V. Ciolfi C. D’Angelo A. Tinelli C. (2021). Psoriasis and diabetes, a dangerous association: evaluation of insulin resistance, lipid abnormalities, and cardiovascular risk biomarkers. Front. Med. 8, 605691. 10.3389/fmed.2021.605691 33834030 PMC8021695

[B21] Brocks T. Fedorchenko O. Schliermann N. Stein A. Moll U. M. Seegobin S. (2017). Macrophage migration inhibitory factor protects from nonmelanoma epidermal tumors by regulating the number of antigen-presenting cells in skin. Faseb J. 31 (2), 526–543. 10.1096/fj.201600860R 27825106 PMC6137604

[B22] Brohem C. A. Cardeal L. B. Tiago M. Soengas M. S. Barros S. B. Maria-Engler S. S. (2011). Artificial skin in perspective: concepts and applications. Pigment. Cell. Melanoma Res. 24 (1), 35–50. 10.1111/j.1755-148X.2010.00786.x 21029393 PMC3021617

[B23] Cai Y. Shen X. Ding C. Qi C. Li K. Li X. (2011). Pivotal role of dermal IL-17-Producing γδ T cells in skin inflammation. Immunity 35 (4), 596–610. 10.1016/j.immuni.2011.08.001 21982596 PMC3205267

[B24] Calandra T. Roger T. (2003). Macrophage migration inhibitory factor: a regulator of innate immunity. Nat. Rev. Immunol. 3 (10), 791–800. 10.1038/nri1200 14502271 PMC7097468

[B25] Carretero G. Puig L. Dehesa L. Carrascosa J. M. Ribera M. Sánchez-Regaña M. (2010). Guidelines on the use of methotrexate in psoriasis. Actas Dermo-Sifiliográficas Engl. Ed. 101 (7), 600–613. 10.1016/s1578-2190(10)70682-x 28709542

[B26] Cetean S. Căinap C. Constantin A. M. Căinap S. Gherman A. Oprean L. (2015). The importance of the granulocyte-colony stimulating factor in oncology. Clujul Med. 88 (4), 468–472. 10.15386/cjmed-531 26732055 PMC4689238

[B27] Chang W.-T. Huang W.-C. Liou C.-J. (2012). Evaluation of the anti-inflammatory effects of phloretin and phlorizin in lipopolysaccharide-stimulated mouse macrophages. Food Chem. 134 (2), 972–979. 10.1016/j.foodchem.2012.03.002 23107715

[B28] Chen C. Shao Y. (2016). Elevated Interleukin-17 levels in patients with newly diagnosed type 2 diabetes mellitus. Biochem. and Physiology Open Access 5 (2), 1–6. 10.4172/2168-9652.1000206

[B29] Chen Z. Tessmer G. Nguyen B. A. Meyer J. Saleem M. Ahmad T. (2025). G-CSF mediates increased renal neutrophils and kidney damage in a psoriasis mouse model. Hypertension 82 (11), 2027–2039. 10.1161/hypertensionaha.125.25111 40905133 PMC12529989

[B30] Cheng J. Kuai D. Y. Zhang L. Yang X. Q. Qiu B. (2012). Psoriasis increased the risk of diabetes: a meta-analysis. Archives Dermatological Res. 304 (2), 119–125. 10.1007/s00403-011-1200-6 22210176

[B31] Clauson P. G. Brismar K. Hall K. Linnarsson R. Grill V. (1998). Insulin-like growth factor-I and insulin-like growth factor binding protein-1 in a representative population of type 2 diabetic patients in Sweden. Scand. J. Clin. Laboratory Investigation 58 (4), 353–360. 10.1080/00365519850186544 9741824

[B32] Crow J. M. (2012). PSORIASIS UNCOVERED. Nature 492 (7429), 550–551. 10.1038/492s50a 23254970

[B33] DeStefano V. Khan S. Tabada A. (2020). Applications of PLA in modern medicine. Eng. Regen. 1, 76–87. 10.1016/j.engreg.2020.08.002 38620328 PMC7474829

[B34] Dludla P. V. Mabhida S. E. Ziqubu K. Nkambule B. B. Mazibuko-Mbeje S. E. Hanser S. (2023). Pancreatic β-cell dysfunction in type 2 diabetes: implications of inflammation and oxidative stress. World J. Diabetes 14 (3), 130–146. 10.4239/wjd.v14.i3.130 37035220 PMC10075035

[B35] Du H. Liu P. Zhu J. Lan J. Li Y. Zhang L. (2019). Hyaluronic acid-based dissolving microneedle patch loaded with methotrexate for improved treatment of psoriasis. ACS Appl. Mater Interfaces 11 (46), 43588–43598. 10.1021/acsami.9b15668 31651148

[B36] El-Komy M. Amin I. Zidan A. Kadry D. Zeid O. A. Shaker O. (2011). Insulin-like growth factor-1 in psoriatic plaques treated with PUVA and methotrexate. J. Eur. Acad. Dermatology Venereol. JEADV 25 (11), 1288–1294. 10.1111/j.1468-3083.2010.03966.x 21241374

[B37] Evans E. A. Sayers S. R. Kodji X. Xia Y. Shaikh M. Rizvi A. (2020). Psoriatic skin inflammation induces a pre-diabetic phenotype *via* the endocrine actions of skin secretome. Mol. Metab. 41, 101047. 10.1016/j.molmet.2020.101047 32599074 PMC7452265

[B38] Fordham J. B. Raza Naqvi A. Nares S. (2014). Leukocyte production of inflammatory mediators is inhibited by the antioxidants phloretin, silymarin, hesperetin, and resveratrol. Mediat. Inflamm. 2014, 938712. 10.1155/2014/938712 24707119 PMC3953573

[B39] Friedrich N. Thuesen B. Jørgensen T. Juul A. Spielhagen C. Wallaschofksi H. (2012). The association between IGF-I and insulin resistance: a general population study in Danish adults. Diabetes Care 35 (4), 768–773. 10.2337/dc11-1833 22374641 PMC3308317

[B40] Fukushima K. Furuya M. Kamimura T. Takimoto-Kamimura M. (2021). Structure of macrophage migration inhibitory factor in complex with methotrexate. Acta Crystallographica. Sect. D. Struct. Biology 77 (Pt 3), 293–299. 10.1107/S2059798321000474 33645533

[B41] Furiati S. C. Catarino J. S. Silva M. V. Silva R. F. Estevam R. B. Teodoro R. B. (2019). Th1, Th17, and treg responses are differently modulated by TNF-α inhibitors and methotrexate in psoriasis patients. Sci. Rep. 9 (1), 7526. 10.1038/s41598-019-43899-9 31101850 PMC6525159

[B42] Gamell C. Bankovacki A. Scalzo-Inguanti K. Sedgmen B. Alhamdoosh M. Gail E. (2023). CSL324, a granulocyte colony-stimulating factor receptor antagonist, blocks neutrophil migration markers that are upregulated in hidradenitis suppurativa. Br. J. Dermatology 188 (5), 636–648. 10.1093/bjd/ljad013 36691791

[B43] Gilet J. Chang Y. Chenivesse C. Legendre B. Vorng H. Duez C. (2009). Role of CCL17 in the generation of cutaneous inflammatory reactions in Hu-PBMC-SCID mice grafted with human skin. J. Invest. Dermatol 129 (4), 879–890. 10.1038/jid.2008.333 19005490

[B44] Girkin J. (2022). Is CC chemokine ligand 17 (TARC) driving disease progression in chronic obstructive pulmonary disease? Am. J. Respir. Cell. Mol. Biol. 66 (4), 358–360. 10.1165/rcmb.2021-0518ed 35133243 PMC8990115

[B45] Goldsmith L. A. Katz S. I. Gilchrest B. A. Paller A. Leffell D. J. Wolf K. (2012). Fitzpatrick's Dermatology in General Medicine. 8th edn, New York: McGraw-Hill Medical.

[B46] Gottlieb A. B. Chao C. Dann F. (2008). Psoriasis comorbidities. J. Dermatological Treat. 19 (1), 5–21. 10.1080/09546630701364768 18273720

[B47] Gowda B. H. J. Ahmed M. G. Hani U. Kesharwani P. Wahab S. Paul K. (2023). Microneedles as a momentous platform for psoriasis therapy and diagnosis: a state-of-the-art review. Int. J. Pharm. 632, 122591. 10.1016/j.ijpharm.2023.122591 36626973

[B48] Gray E. E. Suzuki K. Cyster J. G. (2011). Cutting edge: identification of a motile IL-17-producing gammadelta T cell population in the dermis. J. Immunol. 186 (11), 6091–6095. 10.4049/jimmunol.1100427 21536803 PMC3098921

[B49] Gude M. F. Frystyk J. Flyvbjerg A. Bruun J. M. Richelsen B. Pedersen S. B. (2012). The production and regulation of IGF and IGFBPs in human adipose tissue cultures. Growth Hormone and IGF Res. 22 (6), 200–205. 10.1016/j.ghir.2012.09.004 23079385

[B50] Halder J. Rath G. Rai V. K. (2022). Cyclosporine coated microneedle for transcutaneous delivery: characterization, *in-vitro* evaluation, and *in vivo* anti-psoriatic efficacy against IMQ induced psoriasis. J. Drug Deliv. Sci. Technol. 73, 103450. 10.1016/j.jddst.2022.103450

[B51] Hamilton J. A. Anderson G. P. (2004). GM-CSF biology. GROWTH Factors 22 (4), 225–231. 10.1080/08977190412331279881 15621725

[B52] Haywood N. J. Slater T. A. Matthews C. J. Wheatcroft S. B. (2019). The insulin like growth factor and binding protein family: novel therapeutic targets in obesity and diabetes. Mol. Metab. 19, 86–96. 10.1016/j.molmet.2018.10.008 30392760 PMC6323188

[B53] Hodak E. Gottlieb A. B. Anzilotti M. Krueger J. G. (1996). The insulin-like growth factor 1 receptor is expressed by epithelial cells with proliferative potential in human epidermis and skin appendages: correlation of increased expression with epidermal hyperplasia. J. Invest. Dermatol 106 (3), 564–570. 10.1111/1523-1747.ep12344044 8648195

[B54] Holm J. G. Thomsen S. F. (2019). Type 2 diabetes and psoriasis: links and risks. Psoriasis-Targets Ther. 9, 1–6. 10.2147/PTT.S159163 30697518 PMC6340647

[B55] Holt R. I. G. Flyvbjerg A. (2024). Textbook of Diabetes. Hoboken, NJ: Wiley-Blackwell.

[B56] Hotamisligil G. S. (2006). Inflammation and metabolic disorders. Nature 444 (7121), 860–867. 10.1038/nature05485 17167474

[B57] Hytti M. Ruuth J. Kanerva I. Bhattarai N. Pedersen M. L. Nielsen C. U. (2023). Phloretin inhibits glucose transport and reduces inflammation in human retinal pigment epithelial cells. Mol. Cell. Biochem. 478 (1), 215–227. 10.1007/s11010-022-04504-2 35771396 PMC9836970

[B58] Janusz I. Lewandowski K. Lukamowicz J. Swiatkowska E. Narbutt J. Zalewska-Janowska A. (2010). Insulin resistance and adiponectin levels in psoriasis patients. Postepy Dermatol. I Alergol. 27 (6), 451–455.

[B59] Jeong H. R. Kim J. Y. Kim S. N. Park J. H. (2018). Local dermal delivery of cyclosporin A, a hydrophobic and high molecular weight drug, using dissolving microneedles. Eur. J. Pharm. Biopharm. 127, 237–243. 10.1016/j.ejpb.2018.02.014 29432892

[B60] Johansen C. Kragballe K. Westergaard M. Henningsen J. Kristiansen K. Iversen L. (2005). The mitogen‐activated protein kinases p38 and ERK1/2 are increased in lesional psoriatic skin. Br. J. Dermatology 152 (1), 37–42. 10.1111/j.1365-2133.2004.06304.x 15656798

[B61] Katakura F. Nishiya K. Wentzel A. S. Hino E. Miyamae J. Okano M. (2019). Paralogs of common carp granulocyte colony-stimulating factor (G-CSF) have different functions regarding development, trafficking and activation of neutrophils. Front. Immunol. 10, 255. 10.3389/fimmu.2019.00255 30837998 PMC6389648

[B62] Kim D. H. Sandoval D. Reed J. A. Matter E. K. Tolod E. G. Woods S. C. (2008). The role of GM-CSF in adipose tissue inflammation. Am. J. Physiol. Endocrinol. Metab. 295 (5), E1038–E1046. 10.1152/ajpendo.00061.2008 18765677 PMC2584818

[B63] Korkmaz E. Friedrich E. E. Ramadan M. H. Erdos G. Mathers A. R. Burak Ozdoganlar O. (2015). Therapeutic intradermal delivery of tumor necrosis factor-alpha antibodies using tip-loaded dissolvable microneedle arrays. Acta Biomater. 24, 96–105. 10.1016/j.actbio.2015.05.036 26093066 PMC8266287

[B64] Krane J. F. Gottlieb A. B. Carter D. M. Krueger J. G. (1992). The insulin-like growth factor I receptor is overexpressed in psoriatic epidermis, but is differentially regulated from the epidermal growth factor receptor. J. Exp. Med. 175 (4), 1081–1090. 10.1084/jem.175.4.1081 1313074 PMC2119176

[B65] Lazic S. E. Clarke-Williams C. J. Munafò M. R. (2018). What exactly is 'N' in cell culture and animal experiments? PLoS Biol. 16 (4), e2005282. 10.1371/journal.pbio.2005282 29617358 PMC5902037

[B66] Lecewicz-Torun B. Chodorowska G. Borowiec M. Wojnowska D. Jazienicka I. Czelej D. (2001). “Granulocyte-macrophage colony stimulating factor in plasma of psoriatic patients,” in 10th Congress of the european-academy-of-dermatology-and-venereology (Munich, Germany).

[B67] LeRoith D. Gavrilova O. (2006). Mouse models created to study the pathophysiology of type 2 diabetes. Int. J. Biochem. Cell. Biol. 38 (5-6), 904–912. 10.1016/j.biocel.2005.01.019 16103004

[B68] Lewis D. A. Travers J. B. Somani A. K. Spandau D. F. (2010). The IGF-1/IGF-1R signaling axis in the skin: a new role for the dermis in aging-associated skin cancer. Oncogene 29 (10), 1475–1485. 10.1038/onc.2009.440 19966862 PMC2837099

[B69] Lim W. S. Teoh S. E. Tang A. S. P. Tan B. J. M. Lee J. Y. Yau C. E. (2024). The effects of anti-TNF-α biologics on insulin resistance and insulin sensitivity in patients with rheumatoid arthritis: an update systematic review and meta-analysis. Diabetes Metab. Syndr. 18 (4), 103001. 10.1016/j.dsx.2024.103001 38604059

[B70] Lotfi N. Thome R. Rezaei N. Zhang G.-X. Rezaei A. Rostami A. (2019). Roles of GM-CSF in the pathogenesis of autoimmune diseases: an update. Front. Immunol. 10, 1265. 10.3389/fimmu.2019.01265 31275302 PMC6593264

[B71] Lüdecke D. (2018). Ggeffects: Tidy data frames of marginal effects from regression models. J. Open Source Softw. 3, 772. 10.21105/joss.00772

[B72] Lüdecke D. Ben Shachar M. Patil I. Waggoner P. Makowski D. (2021). Performance: an R package for assessment, comparison and testing of statistical models. J. Open Source Softw. 6, 3139. 10.21105/joss.03139

[B73] Maedler K. Sergeev P. Ris F. Oberholzer J. Joller-Jemelka H. I. Spinas G. A. (2002). Glucose-induced beta cell production of IL-1beta contributes to glucotoxicity in human pancreatic islets. J. Clin. Invest. 110 (6), 851–860. 10.1172/JCI15318 12235117 PMC151125

[B74] Marrakchi S. Puig L. (2022). Pathophysiology of generalized pustular psoriasis. Am. J. Clin. Dermatol 23 (Suppl. 1), 13–19. 10.1007/s40257-021-00655-y PMC8801405 35061228

[B75] Martin G. Guérard S. Fortin M. M. Rusu D. Soucy J. Poubelle P. E. (2012). Pathological crosstalk *in vitro* between T lymphocytes and lesional keratinocytes in psoriasis: necessity of direct cell-to-cell contact. Lab. Invest. 92 (7), 1058–1070. 10.1038/labinvest.2012.69 22525430

[B76] Matos T. R. O'Malley J. T. Lowry E. L. Hamm D. Kirsch I. R. Robins H. S. (2017). Clinically resolved psoriatic lesions contain psoriasis-specific IL-17-producing αβ T cell clones. J. Clin. Invest. 127 (11), 4031–4041. 10.1172/JCI93396 28945199 PMC5663366

[B77] Matthews D. R. Hosker J. P. Rudenski A. S. Naylor B. A. Treacher D. F. Turner R. C. (1985). Homeostasis model assessment: insulin resistance and β-cell function from fasting plasma glucose and insulin concentrations in man. Diabetologia 28 (7), 412–419. 10.1007/BF00280883 3899825

[B78] Maxwell R. A. Eckhardt S. B. (1990). “Methotrexate,” in Drug Discovery: A Casebook and Analysis. Editors Maxwell R. A. Eckhardt S. B. (Totowa, NJ: Humana Press), 249–263.

[B79] Mazo V. K. Sidorova Y. S. Zorin S. N. Kochetkova A. A. (2016). Streptozotocin induced diabetes rat models. Vopr. Pitaniia 85 (4), 14–21. 29381015

[B80] McGinley A. M. Sutton C. E. Edwards S. C. Leane C. M. DeCourcey J. Teijeiro A. (2020). Interleukin-17A serves a priming role in autoimmunity by recruiting IL-1β-Producing myeloid cells that promote pathogenic T cells. Immunity 52 (2), 342–356.e6. 10.1016/j.immuni.2020.01.002 32023490

[B81] Mercurio L. Lulli D. Mascia F. Dellambra E. Scarponi C. Morelli M. (2020). Intracellular insulin-like growth factor binding protein 2 (IGFBP2) contributes to the senescence of keratinocytes in psoriasis by stabilizing cytoplasmic p21. Aging (Albany NY) 12 (8), 6823–6851. 10.18632/aging.103045 32302288 PMC7202509

[B82] Mestas J. Hughes C. C. (2004). Of mice and not men: differences between mouse and human immunology. J. Immunol. 172 (5), 2731–2738. 10.4049/jimmunol.172.5.2731 14978070

[B83] Minh H. V. Tien H. A. Sinh C. T. Thang D. C. Chen C. H. Tay J. C. (2021). Assessment of preferred methods to measure insulin resistance in Asian patients with hypertension. J. Clin. Hypertens. (Greenwich) 23 (3), 529–537. 10.1111/jch.14155 33415834 PMC8029536

[B84] Miura H. Sano S. Higashiyama M. Yoshikawa K. Itami S. (2000). Involvement of insulin-like growth factor-I in psoriasis as a paracrine growth factor: dermal fibroblasts play a regulatory role in developing psoriatic lesions. Arch. Dermatol Res. 292 (12), 590–597. 10.1007/s004030000188 11214819

[B85] Moawad F. Ruel Y. Rezaei N. Alsarraf J. Pichette A. Legault J. (2024). Microneedles with implantable tip-accumulated therapeutics for the long-term management of psoriasis. Small 20, e2405927. 10.1002/smll.202405927 39375985 PMC11657035

[B86] Moawad F. Ruel Y. Pouliot R. Brambilla D. (2025). Microneedle technology in psoriasis management: mechanistic insights, technological innovation, clinical progress, and challenges. Adv. Healthc. Mater 15, e04294. 10.1002/adhm.202504294 41334788 PMC12988575

[B87] Molnar V. Garai J. (2005). Plant-derived anti-inflammatory compounds affect MIF tautomerase activity. Int. Immunopharmacology 5 (5), 849–856. 10.1016/j.intimp.2004.12.017 15778121

[B88] Morand E. F. Leech M. Weedon H. Metz C. Bucala R. Smith M. D. (2002). Macrophage migration inhibitory factor in rheumatoid arthritis: clinical correlations. Rheumatol. Oxf. Engl. 41 (5), 558–562. 10.1093/rheumatology/41.5.558 12011381

[B89] Mosca M. Hong J. Hadeler E. Hakimi M. Liao W. Bhutani T. (2021). The role of IL-17 cytokines in psoriasis. Immunotargets Ther. 10, 409–418. 10.2147/itt.s240891 34853779 PMC8627853

[B90] Nassar A. A. Bakr N. M. Elyousefi E. H. I. Elkholy B. M. Fawzy M. M. (2022). Serum immunoglobulin E and Interleukin-17 levels in patients with chronic plaque psoriasis: a case-control study. J. Cosmetic Dermatology 21 (11), 6377–6384. 10.1111/jocd.15299 35957511

[B91] National Health Service (2023). How and when to Take Methotrexate. Available online at: https://www.nhs.uk/medicines/methotrexate/how-and-when-to-take-methotrexate/ (Accessed June 06, 2025).

[B92] Ning Y. Schuller A. G. P. Bradshaw S. Rotwein P. Ludwig T. Frystyk J. (2006). Diminished growth and enhanced glucose metabolism in triple knockout mice containing mutations of insulin-like growth factor binding Protein-3, -4, and -5. Mol. Endocrinol. 20 (9), 2173–2186. 10.1210/me.2005-0196 16675541

[B93] Ogawa E. Sato Y. Minagawa A. Okuyama R. (2018). Pathogenesis of psoriasis and development of treatment. J. Dermatol 45 (3), 264–272. 10.1111/1346-8138.14139 29226422

[B94] Okuda A. Kubota M. Sawada M. Koishi S. Kataoka A. Bessho R. (1996). Methotrexate inhibits superoxide production and chemotaxis in neutrophils activated by granulocyte colony-stimulating factor. J. Cellular Physiology 168 (1), 183–187. 10.1002/(SICI)1097-4652(199607)168:1<183::AID-JCP22>3.0.CO;2-7 8647914

[B95] Oliveira Mde F. Rocha Bde O. Duarte G. V. (2015). Psoriasis: classical and emerging comorbidities. Bras Dermatol 90 (1), 9–20. 10.1590/abd1806-4841.20153038 25672294 PMC4323693

[B96] Ordelheide A. M. Gommer N. Böhm A. Hermann C. Thielker I. Machicao F. (2016). Granulocyte colony-stimulating factor (G-CSF): a saturated fatty acid-induced myokine with insulin-desensitizing properties in humans. Mol. Metab. 5 (4), 305–316. 10.1016/j.molmet.2016.02.001 27069870 PMC4812007

[B97] Perera G. K. Di Meglio P. Nestle F. O. (2012). Psoriasis. Annu. Rev. Pathol. 7, 385–422. 10.1146/annurev-pathol-011811-132448 22054142

[B98] Polic M. V. Miskulin M. Smolic M. Kralik K. Miskulin I. Berkovic M. C. (2018). Psoriasis Severity-A risk factor of insulin resistance independent of metabolic syndrome. Int. J. Environ. Res. Public Health 15 (7), 1–7. 10.3390/ijerph15071486 30011841 PMC6069377

[B99] Posit Team (2025). Rstudio: Integrated Development Environment for R. Posit Software, PBC, Boston, MA. Available online at: http://www.posit.co/ (Accessed January 07, 2026).

[B100] R Core Team (2024). R: A Language and Environment for Statistical Computing. R Foundation for Statistical Computing, Vienna, Austria. Available online at: https://www.R-project.org/ (Accessed November 04, 2025).

[B101] Ramadon D. McCrudden M. T. C. Courtenay A. J. Donnelly R. F. (2022). Enhancement strategies for transdermal drug delivery systems: current trends and applications. Drug Deliv. Transl. Res. 12 (4), 758–791. 10.1007/s13346-021-00909-6 33474709 PMC7817074

[B102] Ramírez-Valle F. Gray E. E. Cyster J. G. (2015). Inflammation induces dermal Vγ4^+^ γδT17 memory-like cells that travel to distant skin and accelerate secondary IL-17–driven responses. Proc. Natl. Acad. Sci. 112 (26), 8046–8051. 10.1073/pnas.1508990112 26080440 PMC4491769

[B103] Rioux G. Simard M. Morin S. Lorthois I. Guérin S. L. Pouliot R. (2021). Development of a 3D psoriatic skin model optimized for infiltration of IL-17A producing T cells: focus on the crosstalk between T cells and psoriatic keratinocytes. Acta Biomater. 136, 210–222. 10.1016/j.actbio.2021.09.018 34547515

[B104] Ruel Y. Moawad F. Alsarraf J. Pichette A. Legault J. Brambilla D. (2024). Antiproliferative and anti-inflammatory effects of the polyphenols phloretin and balsacone C in a coculture of T cells and psoriatic keratinocytes. Int. J. Mol. Sci. 25 (11), 5639. 10.3390/ijms25115639 38891824 PMC11171971

[B105] Ruel Y. Moawad F. Jean E. Nadeau C. Alsarraf J. Pichette A. (2025). Toward long-acting psoriasis therapy with phloretin-loaded microneedle patches: insights from *in vitro* patient-derived skin models. ACS Pharmacol. and Transl. Sci. 8 (9), 3221–3239. 10.1021/acsptsci.5c00361 40969878 PMC12441853

[B106] Savastano S. Balato N. Gaudiello F. Di Somma C. Brancato V. Colao A. (2011). Insulin-like Growth Factor-1, Psoriasis, and Inflammation: a Ménage à Trois? Eur. J. Inflamm. 9 (3), 277–283. 10.1177/1721727x1100900308

[B107] Schön M. P. Manzke V. Erpenbeck L. (2021). Animal models of psoriasis—highlights and drawbacks. J. Allergy Clin. Immunol. 147 (2), 439–455. 10.1016/j.jaci.2020.04.034 32560971

[B108] Sharma M. Chan H. K. Lavilla C. A. Jr. Uy M. M. Froemming G. R. A. Okechukwu P. N. (2023). Induction of a single dose of streptozotocin (50 mg) in rat model causes insulin resistance with type 2 diabetes mellitus. Fundam. Clin. Pharmacol. 37 (4), 769–778. 10.1111/fcp.12892 36905079

[B109] Shelke V. Kale A. Kulkarni Y. A. Gaikwad A. B. (2024). Phloretin: a comprehensive review of its potential against diabetes and associated complications. J. Pharmacy Pharmacology 76 (3), 201–212. 10.1093/jpp/rgae010 38243397

[B110] Shen X. Zhou N. Mi L. Hu Z. Wang L. Liu X. (2017). Phloretin exerts hypoglycemic effect in streptozotocin-induced diabetic rats and improves insulin resistance *in vitro* . Drug Des. Devel Ther. 11, 313–324. 10.2147/DDDT.S127010 28223777 PMC5304989

[B111] Shen X. Wang L. Zhou N. Gai S. Liu X. Zhang S. (2020). Beneficial effects of combination therapy of phloretin and metformin in streptozotocin-induced diabetic rats and improved insulin sensitivity *in vitro* . Food and Funct. 11 (1), 392–403. 10.1039/c9fo01326a 31821397

[B112] Shimizu T. Nakamura H. Abe R. Watanabe H. Ohkawara A. Shimizu H. (2001). High macrophage migration inhibitory factor (MIF) serum levels associated with extended psoriasis. J. Investigative Dermatology 116 (6), 989–990. 10.1046/j.0022-202x.2001.01366.x 11407993

[B113] Spurlock C. F. III Gass H. M. I. V. Bryant C. J. Wells B. C. Olsen N. J. Aune T. M. (2015). Methotrexate-mediated inhibition of nuclear factor κB activation by distinct pathways in T cells and fibroblast-like synoviocytes. Rheumatology 54 (1), 178–187. 10.1093/rheumatology/keu279 25118313 PMC4269792

[B114] Steinhoff M. Meinhardt A. Steinhoff A. Gemsa D. Bucala R. Bacher M. (1999). Evidence for a role of macrophage migration inhibitory factor in psoriatic skin disease. Br. J. Dermatology 141 (6), 1061–1066. 10.1046/j.1365-2133.1999.03206.x 10606853

[B115] Stojanović I. Cvjetićanin T. Lazaroski S. Stosić-Grujicić S. Miljković D. (2009). Macrophage migration inhibitory factor stimulates interleukin-17 expression and production in lymph node cells. Immunology 126 (1), 74–83. 10.1111/j.1365-2567.2008.02879.x 18624729 PMC2632697

[B116] Stošić-Grujičić S. Saksida T. Miljković Đ. Stojanović I. (2020). MIF and insulin: lifetime companions from common genesis to common pathogenesis. Cytokine 125, 154792. 10.1016/j.cyto.2019.154792 31400637

[B117] Takematsu H. Tagami H. (1990). Granulocyte-macrophage colony-stimulating factor in psoriasis. Dermatologica 181 (1), 16–20. 10.1159/000247852 2394298

[B118] Global Psoriasis Atlas (2026). The Third Edition of the Global Psoriasis Atlas. Available online at: https://www.globalpsoriasisatlas.org/ (Accessed February 02, 2026).

[B119] Wang Y. Qin B. Xia G. Choi S. H. (2021). FDA's poly (lactic-co-glycolic acid) research program and regulatory outcomes. Aaps J. 23 (4), 92. 10.1208/s12248-021-00611-y 34189655

[B120] Wang L. Pan J. Jin H. (2022). Profiling and multivariate analysis of serum cytokines in patients with generalized pustular psoriasis. Eur. J. Inflamm. 20, 20587392221076450. 10.1177/20587392221076450

[B121] Wang Z. Y. Zhao Z. Q. Sheng Y. J. Chen K. J. Chen B. Z. Guo X. D. (2024). Dual-action psoriasis therapy: antiproliferative and immunomodulatory effects *via* self-locking microneedles. Adv. Sci. (Weinh) 11 (48), e2409359. 10.1002/advs.202409359 39473371 PMC11672289

[B122] Warren R. B. Weatherhead S. C. Smith C. H. Exton L. S. Mohd Mustapa M. F. Kirby B. (2016). British association of dermatologists' guidelines for the safe and effective prescribing of methotrexate for skin disease 2016. Br. J. Dermatol 175 (1), 23–44. 10.1111/bjd.14816 27484275

[B123] Wieser V. Moschen A. R. Tilg H. (2013). Inflammation, cytokines and insulin resistance: a clinical perspective. Arch. Immunol. Ther. Exp. Warsz. 61 (2), 119–125. 10.1007/s00005-012-0210-1 23307037

[B124] Williamson G. (2022). Effects of polyphenols on glucose-induced metabolic changes in healthy human subjects and on glucose transporters. Mol. Nutr. and Food Res. 66 (21), 2101113. 10.1002/mnfr.202101113 35315210 PMC9788283

[B125] Wu D. Shou X. Yu Y. Wang X. Chen G. Zhao Y. (2022). Biologics-loaded photothermally dissolvable hyaluronic acid microneedle patch for psoriasis treatment. Adv. Funct. Mater. 32 (47), 2205847. 10.1002/adfm.202205847

[B126] Yoshiki R. Kabashima K. Honda T. Nakamizo S. Sawada Y. Sugita K. (2014). IL-23 from langerhans cells is required for the development of imiquimod-induced psoriasis-like dermatitis by induction of IL-17A-Producing γδ T cells. J. Investigative Dermatology 134 (7), 1912–1921. 10.1038/jid.2014.98 24569709

[B127] Zeitler P. S. Nadeau K. J. (2020). Insulin Resistance: Childhood Precursors of Adult Disease. Cham: Humana Press.

[B128] Zhang M. Su W. Deng J. Zhai B. Zhu G. Gao R. (2025). Multi-ancestry genome-wide meta-analysis with 472,819 individuals identifies 32 novel risk loci for psoriasis. J. Transl. Med. 23 (1), 133. 10.1186/s12967-024-06015-8 39885523 PMC11783861

