# Supplementary material for: A tissue-engineered human psoriatic skin model: targeting inflammation and glucose metabolism dysregulation in psoriasis using microneedle patches
Source: Front Mol Biosci. 2026 Jun 9;13:1830240. doi: 10.3389/fmolb.2026.1830240 (PMC13286830; doi:10.3389/fmolb.2026.1830240)
Supplement: Supplementary file 1 [file DataSheet1.pdf]

Supplementary materials

A Tissue-Engineered Human Psoriatic Skin Model: Targeting Inflammation and Glucose Metabolism Dysregulation in Psoriasis Using Microneedle Patches

*Yasmine Ruel [1,2], Fatma Moawad [3], Sergio Cortez Ghio [4], Davide Brambilla [3,5] and Roxane Pouliot [1,2,]**

[1]Faculté de Pharmacie, Université Laval, Québec City, QC G1V 0A6, Canada

[2]Centre de Recherche en Organogénèse Expérimentale de l'Université Laval/LOEX, Axe Médecine Régénératrice, Centre de Recherche du CHU de Québec-Université Laval, Québec City, QC G1J 2Z4, Canada; yasmine.ruel.1@ulaval.ca (Y.R.).

[3]Faculté de pharmacie, Université de Montréal, QC H3C 3J7, Canada; fatma.moawad@umontreal.ca (F.M.); davide.brambilla@umontreal.ca (D.B.).

[4] In Silico Data Science, Québec City, QC G1E 6P3, Canada; sergio.cortez.ghio@is-data.science (S.C.G.).

[5] School of Pharmaceutical Sciences, University of Geneva, 1211, Geneva, Switzerland

*Correspondence: Roxane.Pouliot@pha.ulaval.ca (R.P.); Tel.: +1-418-525-4444 (ext. 61706)

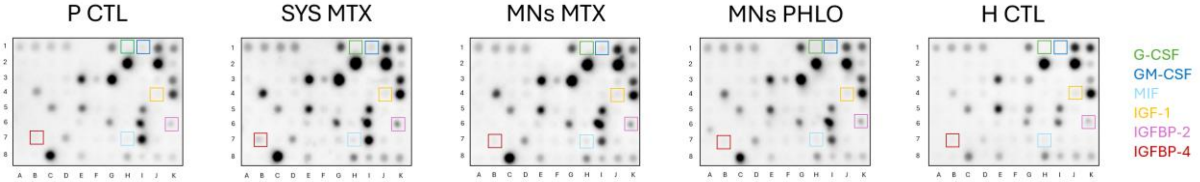

Figure S1. Protein array analysis of 80 secreted proteins was performed using the Human Cytokine Array C5 from RayBiotech, on culture supernatants from psoriatic and healthy skin substitutes. Psoriatic control (P CTL) and healthy control (H CTL) were produced. Treatments were applied to psoriatic skin substitutes; systemic-like methotrexate (SYS MTX), methotrexate-loaded microneedle patches (MNs MTX), and phloretin-loaded microneedle patches (MNs PHLO). To assess the inflammatory response, cytokines altered significantly following the treatments were highlighted; granulocyte colony-stimulating factor (G-CSF), granulocyte-macrophage colony-stimulating factor (GM-CSF), and macrophage migration inhibitory factor (MIF). To complete the analysis of the glucose pathway in psoriasis using tissue-engineered human psoriatic skin substitutes, insulin-like growth factor 1 (IGF-1), and its most abundantly expressed binding proteins in the supernatants, insulin-like growth factor binding protein 2 (IGFBP-2), and insulin-like growth factor binding protein 4 (IGFBP-4), were also analyzed. One membrane per condition is shown.

Table S1. Information about the Donors

Type of Donor	Population	Age	Sex	Region of the biopsy	Percentage of body surface involved	PASI Score
With Psoriasis	PSO1	39	Male	NA	NA	17
	PSO2	65	Male	NA	NA	11
	PSO3	36	Female	Back	5% of the body	NA
Healthy	H1	29	Female	breast	N/A	N/A
	H2	42	Female	breast	N/A	N/A
	H3	47	Female	breast	N/A	N/A

NA: data not available, N/A = Not Applicable

Table S2. Inflammatory Response — Mean Cytokine Levels Measured by the Integrated Density of Array Blots

Condition	G-CSF	GM-CSF	MIF
**P CTL**	382.695 ± 221.848	137.364 ± 158.908	196.331 ± 52.572
**SYS MTX**	109.749 ± 53.851	142.062 ± 126.471	129.722 ± 32.505
**MNs MTX**	125.249 ± 115.669	127.949 ± 133.309	181.868 ± 51.160
**MNs PHLO**	147.741 ± 55.203	56.200 ± 64.681	122.161 ± 23.230
**H CTL**	186.055 ± 164.576	15.209 ± 18.298	172.658 ± 56.184

Data ± Standard deviation

Table S3. Inflammatory Response (Cytokine Array) — Statistical Analysis Using a Mixed-Effects Linear Model (from Figure 1)

Mixed-Effects G-CSF

Condition	Estimate	Confidence interval	
		2.50%	97.50%
SYS MTX	−272.946	−416.365	−129.527
MNs MTX	−257.446	−400.865	−114.027
MNs PHLO	−234.954	−378.373	−91.535
H CTL	−196.640	−426.200	32.919

P CTL is the reference group

Mixed-Effects GM-CSF

Condition	Estimate	Confidence interval	
		2.50%	97.50%
SYS MTX	4.698	−66.670	76.067
MNs MTX	−9.415	−80.783	61.954
MNs PHLO	−81.164	−152.532	−9.795
H CTL	−122.155	−276.920	32.61

P CTL is the reference group

Mixed-Effects MIF

Condition	Estimate	Confidence interval	
		2.50%	97.50%
SYS MTX	−66.609	−117.255	−15.963
MNs MTX	−14.463	−65.109	36.183
MNs PHLO	−74.170	−124.816	−23.524
H CTL	−23.673	−98.688	51.341

P CTL is the reference group

Table S4. IL-17A Concentration in Culture Supernatants (pg/mL) — Statistical Analysis Using a Mixed-Effects Linear Model (from Figure 2)

Condition	Estimate	Confidence interval	
		2.5%	97.5%
**SYS MTX**	0.896	−0.694	2.485
**MNs MTX**	−1.260	−2.850	0.329
**MNs PHLO**	−1.590	−3.179	−0.001
**H CTL**	−2.102	−6.278	2.075

P CTL is the reference group

Table S5. Mean Percentages of Glucose and Insulin Uptake from the Culture Medium by Cells in Healthy and Psoriatic Skin Substitute Cultures

Condition	Glucose Uptake (%)	Insulin Uptake (%)
**P CTL**	4.808 ± 1.141	8.904 ± 10.136
**SYS MTX**	4.628 ± 2.088	9.315 ± 7.541
**MNs MTX**	4.496 ± 1.001	8.785 ± 8.876
**MNs PHLO**	4.530 ± 1.028	11.239 ± 8.658
**H CTL**	39.103 ± 44.978	14.665 ± 6.636

Data ± Standard deviation

Table S6. Percentage of Glucose and Insulin Uptake from the Culture Medium — Statistical Analysis Using a Fixed-Effects Linear Model (from Figure 3a,c)

Fixed-Effects: Glucose

Condition	Estimate	Confidence interval	
		2.5%	97.5%
**H CTL**	34.295	2.502	66.088

P CTL is the reference group

Fixed-Effects: Insulin

Condition	Estimate	Confidence interval	
		2.50%	97.50%
**H CTL**	5.761	-2.800	14.322

P CTL is the reference group

Table S7. Percentage of Glucose and Insulin Uptake from the Culture Medium — Statistical Analysis Using a Mixed-Effects Linear Model (from Figure 3b,d)

Mixed-Effects: Glucose

Condition	Estimate	Confidence interval	
		2.50%	97.50%
**SYS MTX**	-0.18	-1.369	1.009
**MNs MTX**	-0.312	-1.501	0.877
**MNs PHLO**	-0.278	-1.467	0.911
**H CTL**	34.295	-24.464	93.054

P CTL is the reference group

Mixed-Effects: Insulin

Condition	Estimate	Confidence interval	
		2.50%	97.50%
**SYS MTX**	0.411	-3.411	4.233
**MNs MTX**	-0.119	-3.941	3.703
**MNs PHLO**	2.335	-1.486	6.157
**H CTL**	5.761	-6.835	18.357

P CTL is the reference group

Table S8. Insulin-Like Growth Factor Signalling — Mean Protein Levels Measured by the Integrated Density of Array Blots

Condition	IGF-1	IGFBP-2	IGFBP-4
**P CTL**	168.189 ± 39.302	763.753 ± 315.518	232.052 ± 38.671
**SYS MTX**	140.747 ± 55.696	887.567 ± 298.963	367.012 ± 188.807
**MNs MTX**	169.946 ± 59.303	909.488 ± 332.960	208.029 ± 102.963
**MNs PHLO**	173.899 ± 65.865	661.938 ± 352.223	139.007 ± 41.080
**H CTL**	232.802 ± 53.876	1280.619 ± 365.137	123.813 ± 43.876

Data ± Standard deviation

Table S9. Insulin-Like Growth Factor Signalling — Statistical Analysis Using a Fixed-Effects Linear Model (from Figure 4a,c,e)

Fixed-Effects: IGF-1

Condition	Estimate	Confidence interval	
		**2.50%**	**97.50%**
**H CTL**	64.631	-42.287	171.513

P CTL is the reference group

Fixed-Effects: IGFBP-2

Condition	Estimate	Confidence interval	
		**2.50%**	**97.50%**
**H CTL**	516.866	-256.690	1290.42

P CTL is the reference group

Fixed-Effects: IGFBP-4

Condition	Estimate	Confidence interval	
		**2.50%**	**97.50%**
**H CTL**	-108.239	-201.990	-14.489

P CTL is the reference group

Table S10. Insulin-Like Growth Factor Signalling — Statistical Analysis Using a Mixed-Effects Linear Model (from Figure 4b,d,f)

Mixed-Effects IGF-1

Condition	Estimate	Confidence interval	
		2.50%	97.50%
SYS MTX	-27.442	-73.110	18.227
MNs MTX	1.757	-43.912	47.426
MNs PHLO	5.710	-39.959	51.379
H CTL	64.613	-23.586	152.812

P CTL is the reference group

Mixed-Effects: IGFBP-2

Condition	Estimate	Confidence interval	
		2.50%	97.50%
SYS MTX	123.814	-70.530	318.158
MNs MTX	145.735	-48.609	340.080
MNs PHLO	-101.815	-296.160	92.529
H CTL	516.866	-33.625	1067.356

P CTL is the reference group

Mixed-Effects: IGFBP-4

Condition	Estimate	Confidence interval	
		2.50%	97,50%
SYS MTX	134.960	-23.088	293.008
MNs MTX	-24.023	-182.071	134.025
MNs PHLO	-93.045	-251.093	65.003
H CTL	-108.239	-270.332	53.853

P CTL is the reference group
